# Supplementary material for: Changes in DNA Methylation and mRNA Expression in Lung Tissue after Long-Term Supplementation with an Increased Dose of Cholecalciferol
Source: Int J Mol Sci. 2023 Dec 29;25(1):464. doi: 10.3390/ijms25010464 (PMC10778667; doi:10.3390/ijms25010464)
Supplement: Supplementary file 1 [file ijms-25-00464-s001.zip › Supplementary material Figure S1.pdf]

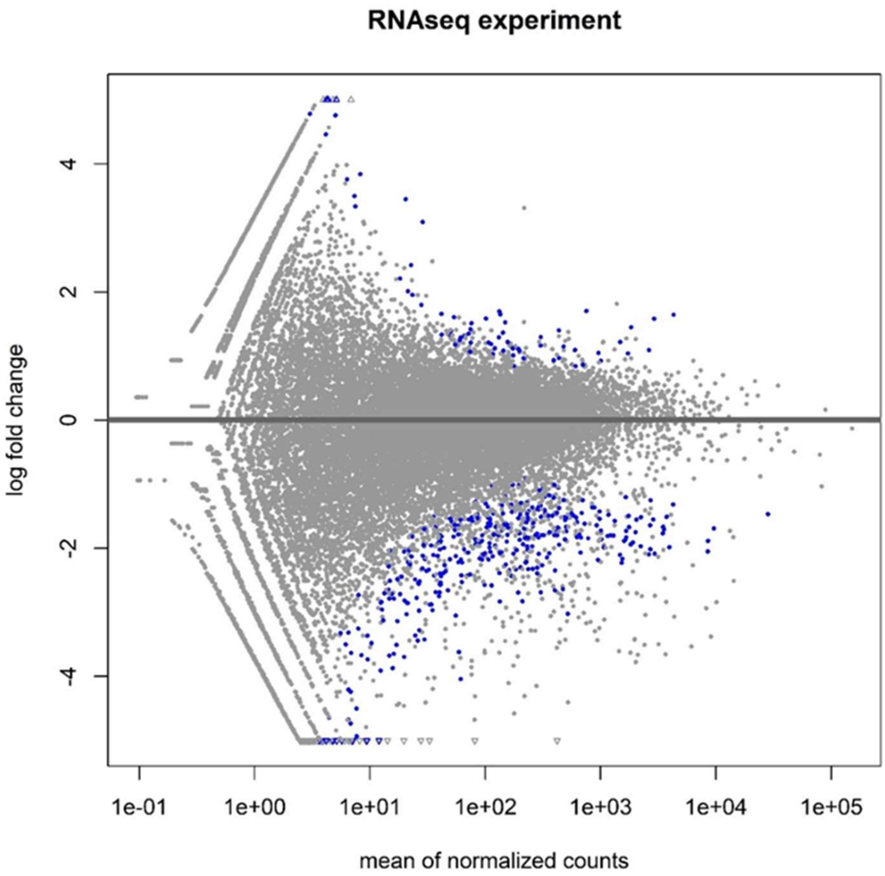

Supplementary Material Figure S1. The level of gene expression changes under the influence of increased dose of cholecalciferol. The graph shows upregulated and downregulated genes (dots). Blue dots indicate genes whose change in expression are statistically significant.
