# Supplementary material for: Changes in DNA Methylation and mRNA Expression in Lung Tissue after Long-Term Supplementation with an Increased Dose of Cholecalciferol
Source: Int J Mol Sci. 2023 Dec 29;25(1):464. doi: 10.3390/ijms25010464 (PMC10778667; doi:10.3390/ijms25010464)
Supplement: Supplementary file 1 [file ijms-25-00464-s001.zip › Supplementary Material Table S1.pdf]

Supplementary Material Table S1. Statistics of RRBS library sequencing results.

Sample	No. of raw reads	No. of filtered reads	% of uniquely mapped reads	No. of uniquely mapped reads
10I	33503565	32846633	63,5	20846474
11II	30100643	29510435	62,6	18459212
12II	27333224	26797279	64,4	17255052
17I	38081824	37335122	64	23891231
18I	28741640	28178079	64,2	18099373
19II	24448049	23968676	65	15577415
20II	30192440	29600432	64,5	19098076
26I	28108431	27557286	63,8	17577519
27II	28756797	28192939	64	18055125
33I	30684315	30082662	65	19554099
34I	30362653	29767307	63,6	18936308
3II	28451110	27893246	64,9	18093212
41I	31913789	31288029	64,3	20132044
44II	34024361	33357217	62	20696372
51II	28699064	28136338	65,9	18528538
9I	30622687	30022243	64,8	19460751
**Average**	**30251537**	**29658370,19**	**64,15625**	**19016300,06**
