# Supplementary material for: Changes in DNA Methylation and mRNA Expression in Lung Tissue after Long-Term Supplementation with an Increased Dose of Cholecalciferol
Source: Int J Mol Sci. 2023 Dec 29;25(1):464. doi: 10.3390/ijms25010464 (PMC10778667; doi:10.3390/ijms25010464)
Supplement: Supplementary file 1 [file ijms-25-00464-s001.zip › Supplementary Material Table S2.pdf]

Supplementary Material Table S2. Changes in methylation identified in animals receiving an increased dose of cholecalciferol.

Ensembl ID	Gene name	qvalue	meth.diff	Consequence
ENSSSCG00000038807	SCAF1	2,08198E-63	-63,52562295	intron_variant
ENSSSCG00000038264	-	1,31241E-93	-60,98862863	intron_variant
ENSSSCG00000024480	SFMBT2	8,13795E-27	-60,07235264	intron_variant
ENSSSCG00000022302	BICRA	1,2568E-48	-54,49175137	intron_variant
ENSSSCG00000016851	OSMR	1,98289E-56	-54,28938356	intron_variant
ENSSSCG00000021874	UNC5C	2,89398E-21	-53,68077551	downstream_gene_variant
ENSSSCG00000027415	WWOX	3,30438E-42	-53,27394209	intron_variant
ENSSSCG00000003115	MEIS3	1,23048E-77	-52,65734266	intron_variant
ENSSSCG00000003255	CACNG8	6,69894E-44	-51,64312039	intron_variant
ENSSSCG00000035414	XXYLT1	1,57291E-23	-51,43870314	intron_variant
ENSSSCG00000010536	CNNM1	5,94097E-84	-50,93593251	intron_variant
ENSSSCG00000005711	NUP214	1,0447E-26	-50,37030506	intron_variant
ENSSSCG00000002806	MMP15	3,0209E-107	-48,66729692	upstream_gene_variant
ENSSSCG00000015729	TSN	1,88904E-66	-48,29693049	intron_variant
ENSSSCG00000006200	CPA6	3,19978E-43	-47,70036322	intron_variant
ENSSSCG00000035020	STK32B	1,13101E-17	-47,67166535	intron_variant
ENSSSCG00000006725	-	9,1427E-22	-47,40634382	intron_variant
ENSSSCG00000001535	TCP11	3,77553E-36	-46,92126851	intron_variant
ENSSSCG00000010757	EBF3	3,17899E-15	-45,74885104	intron_variant
ENSSSCG00000003229	ETFB	1,57163E-31	-45,51724138	intron_variant
ENSSSCG00000008724	ACOX3	1,05488E-27	-45,51229508	intron_variant
ENSSSCG00000002857	TDRD12	2,14206E-20	-45,12396694	upstream_gene_variant
ENSSSCG00000002314	SMOC1	3,90164E-31	-45,11771611	downstream_gene_variant
ENSSSCG00000039372	EMC10	4,33022E-22	-45,03745318	downstream_gene_variant
ENSSSCG00000017139	RPTOR	6,39448E-23	-44,72929668	intron_variant
ENSSSCG00000009222	SPARCL1	1,10817E-23	-44,61726713	intron_variant
ENSSSCG00000035045	DOCK1	1,50577E-20	-44,59160419	intron_variant
ENSSSCG00000013078	-	1,30498E-22	-44,43319838	upstream_gene_variant
ENSSSCG00000023172	-	5,20721E-26	-44,27952999	intron_variant
ENSSSCG00000042741	-	2,98568E-22	-44,19014659	intron_variant,non_coding_transcript_variant
ENSSSCG00000035136	SDCCAG8	4,571E-21	-43,98384983	intron_variant
ENSSSCG00000031706	-	3,76991E-71	-43,78480043	intron_variant
ENSSSCG00000055222	-	5,68555E-25	-43,73229796	intron_variant,non_coding_transcript_variant
ENSSSCG00000009308	LNX2	9,41121E-30	-43,57774503	downstream_gene_variant
ENSSSCG00000006054	ATP6V1C1	4,10816E-24	-43,49868044	intron_variant
ENSSSCG00000015432	ATXN7L1	1,60885E-35	-43,44868382	intron_variant
ENSSSCG00000008943	SLC4A4	6,8031E-65	-43,44494233	intron_variant
ENSSSCG00000001422	C2	8,51571E-56	-43,1316409	intron_variant
ENSSSCG00000017136	TBCD	3,18604E-14	-43,09024309	intron_variant
ENSSSCG00000006355	APOA2	5,65426E-32	-43,08470997	downstream_gene_variant
ENSSSCG00000023738	COG3	9,40686E-26	-42,92590154	downstream_gene_variant
ENSSSCG00000043505	-	6,05905E-19	-42,36727222	intron_variant,non_coding_transcript_variant

ENSSSCG00000009208	HERC3	2,18293E-18	-42,3573073	intron_variant
ENSSSCG00000033733	-	1,69532E-19	-42,24805521	downstream_gene_variant
ENSSSCG00000034286	-	1,03497E-17	-42,18039547	intron_variant
ENSSSCG00000009007	TMEM131L	2,84433E-48	-41,96142611	intron_variant
ENSSSCG00000007574	SDK1	7,07101E-46	-41,8230563	intron_variant
ENSSSCG00000017475	RARA	9,23395E-37	-41,77719115	upstream_gene_variant
ENSSSCG00000028979	UVRAG	1,57654E-24	-41,66289984	intron_variant
ENSSSCG00000025984	TTLL5	3,047E-109	-41,62929764	intron_variant
ENSSSCG00000023371	EHD2	2,66812E-52	-41,57018813	intron_variant
ENSSSCG00000017018	TENM2	5,53793E-13	-41,45384411	intron_variant
ENSSSCG00000013303	ABTB2	4,88539E-17	-41,30952381	intron_variant
ENSSSCG00000016411	NOM1	1,26095E-21	-41,23435352	intron_variant
ENSSSCG00000024954	FGF1	1,77038E-34	-41,07444091	intron_variant
ENSSSCG00000012993	SLC25A45	1,33605E-26	-40,96385542	downstream_gene_variant
ENSSSCG00000021624	-	4,36547E-34	-40,9249455	upstream_gene_variant
ENSSSCG00000010585	ACTR1A	3,636E-108	-40,83956972	intron_variant
ENSSSCG00000016491	DENND11	6,87819E-16	-40,63012905	intron_variant
ENSSSCG00000009389	TRIM13	1,33184E-80	-40,55898068	intron_variant
ENSSSCG00000038417	LRRC8D	4,34049E-10	-40,53907559	intron_variant
ENSSSCG00000001577	TBC1D22B	3,5204E-14	-40,33015296	intron_variant
ENSSSCG00000020702	SENP6	2,1242E-09	-40,30501089	intron_variant
ENSSSCG00000036887	CLPB	4,75027E-08	-40,15643447	intron_variant
ENSSSCG00000026661	PTPRN2	5,14002E-21	-40,03789378	intron_variant
ENSSSCG00000012835	MUC6	1,46951E-16	-40,00666223	intron_variant
ENSSSCG00000036772	CAMTA1	4,78495E-10	-39,96321839	intron_variant
ENSSSCG00000022668	ZFP1	1,86266E-37	-39,77740534	intron_variant
ENSSSCG00000008344	ARHGAP25	1,01837E-59	-39,56920553	upstream_gene_variant
ENSSSCG00000042172	-	2,05834E-59	-39,35537802	intron_variant,non_coding_transcript_variant
ENSSSCG00000000024	PARVB	1,09846E-09	-39,16312057	intron_variant
ENSSSCG00000039713	-	6,45723E-39	-39,15305494	downstream_gene_variant
ENSSSCG00000025052	BPIFB6	4,81319E-16	-38,86908374	upstream_gene_variant
ENSSSCG00000028802	-	1,71018E-20	-38,72211974	upstream_gene_variant
ENSSSCG00000047619	-	2,60234E-19	-38,66196925	intron_variant,non_coding_transcript_variant
ENSSSCG00000016846	WDR70	2,20769E-10	-38,66098081	intron_variant
ENSSSCG00000003398	UBE4B	5,87847E-14	-38,639866	intron_variant
ENSSSCG00000000138	PVALB	1,0691E-14	-38,49889458	intron_variant
ENSSSCG00000016338	PER2	2,83026E-21	-38,33981911	intron_variant
ENSSSCG00000002868	PEPD	4,04485E-21	-38,18851205	intron_variant
ENSSSCG00000021818	ENOX1	1,13959E-11	-38,18181818	intron_variant
ENSSSCG00000032684	BOK	9,2408E-128	-37,97858833	upstream_gene_variant
ENSSSCG00000053835	-	6,16495E-18	-37,93103448	intron_variant
ENSSSCG00000021731	WWC2	1,07191E-12	-37,82212353	intron_variant
ENSSSCG00000033497	-	5,80758E-08	-37,79776373	intron_variant
ENSSSCG00000033082	HGS	2,38478E-10	-37,71228771	splice_region_variant,synonymous_variant
ENSSSCG00000014436	ARHGEF37	1,46215E-17	-37,57409941	intron_variant
ENSSSCG00000029519	CIAPIN1	3,4107E-108	-37,53214534	intron_variant

ENSSSCG00000005617	STXBP1	2,03085E-10	-37,5	intron_variant
ENSSSCG00000046219	-	5,70433E-16	-37,47540984	upstream_gene_variant
ENSSSCG00000037336	GID4	5,87157E-17	-37,37750173	downstream_gene_variant
ENSSSCG00000017790	TAOK1	3,52383E-38	-37,22978975	intron_variant
ENSSSCG00000026894	NFE2L3	8,86752E-29	-37,15744342	upstream_gene_variant
ENSSSCG00000015720	BIN1	6,87146E-15	-36,996337	intron_variant
ENSSSCG00000034086	ATP5F1C	2,02657E-13	-36,95294762	downstream_gene_variant
ENSSSCG00000002370	AREL1	9,04008E-11	-36,84196347	intron_variant
ENSSSCG00000028867	PPIL4	8,09074E-65	-36,80111098	intron_variant
ENSSSCG00000026068	GRHL1	1,30375E-23	-36,77544348	intron_variant
ENSSSCG00000056477	-	3,26208E-17	-36,77477477	non_coding_transcript_exon_variant
ENSSSCG00000031957	RIMS2	4,63468E-20	-36,64285714	intron_variant
ENSSSCG00000010701	BTBD16	5,2629E-139	-36,41072735	intron_variant
ENSSSCG00000002508	SETD3	3,88266E-09	-36,39966782	downstream_gene_variant
ENSSSCG00000012836	AP2A2	6,871E-10	-36,32034632	intron_variant
ENSSSCG00000062980	-	1,48001E-15	-36,27986822	downstream_gene_variant
ENSSSCG00000021145	AP1S1	1,67223E-50	-36,23648479	upstream_gene_variant
ENSSSCG00000034364	SPECC1	1,1538E-15	-36,18595825	intron_variant
ENSSSCG00000007244	POFUT1	6,1934E-14	-36,15886536	downstream_gene_variant
ENSSSCG00000026746	-	2,99705E-14	-36,14478114	downstream_gene_variant
ENSSSCG00000030396	SETD7	2,30173E-13	-36,13258601	intron_variant
ENSSSCG00000038977	RSBN1L	9,521E-12	-36,10325015	intron_variant
ENSSSCG00000006039	LRP12	6,83591E-17	-36,05836284	missense_variant
ENSSSCG00000055606	PLK4	2,65318E-19	-35,98421927	intron_variant
ENSSSCG00000027229	B3GNTL1	2,52318E-15	-35,96265821	intron_variant
ENSSSCG00000032381	-	1,64198E-11	-35,76236465	intron_variant
ENSSSCG00000014876	MYO7A	9,65466E-12	-35,69000288	intron_variant
ENSSSCG00000042107	-	2,35932E-77	-35,57068915	intron_variant,non_coding_transcript_variant
ENSSSCG00000046010	-	1,00023E-12	-35,43268283	downstream_gene_variant
ENSSSCG00000035456	WWC1	3,14809E-33	-35,37424454	intron_variant
ENSSSCG00000046358	SNRNP48	2,6049E-19	-35,3514487	intron_variant
ENSSSCG00000017125	FOXK2	1,92405E-07	-35,31949627	intron_variant
ENSSSCG00000013258	HARBI1	6,97076E-22	-35,28451861	intron_variant
ENSSSCG00000034723	PYCR1	9,71665E-25	-35,24398211	upstream_gene_variant
ENSSSCG00000007681	CUX1	6,60348E-23	-35,24202522	intron_variant
ENSSSCG00000010816	TGFB2	1,34453E-15	-35,0591716	intron_variant
ENSSSCG00000015645	-	1,03677E-24	-34,92056273	downstream_gene_variant
ENSSSCG00000055558	-	1,45819E-07	-34,89952719	intron_variant
ENSSSCG00000057855	-	6,86821E-12	-34,81285311	non_coding_transcript_exon_variant
ENSSSCG00000038731	STX18	2,70201E-11	-34,76760239	intron_variant
ENSSSCG00000029227	LDB2	2,36146E-12	-34,68307507	intron_variant
ENSSSCG00000026680	KCND3	4,449E-23	-34,58463677	intron_variant
ENSSSCG00000059376	EEF1AKMT1	1,8417E-14	-34,52213014	upstream_gene_variant
ENSSSCG00000013174	CTNND1	1,07414E-23	-34,50692334	intron_variant
ENSSSCG00000011234	CMTM7	2,82821E-20	-34,48545511	downstream_gene_variant
ENSSSCG00000047692	-	2,36065E-35	-34,34299877	upstream_gene_variant

ENSSSCG00000048017	-	6,88662E-14	-34,30170576	non_coding_transcript_exon_variant
ENSSSCG00000021060	RCBTB1	2,84567E-19	-34,2823121	intron_variant
ENSSSCG00000006594	SMCP	4,89936E-13	-34,25886865	5_prime_UTR_variant
ENSSSCG00000024399	EVC2	3,06018E-17	-34,24369748	intron_variant
ENSSSCG00000009393	EBPL	4,24982E-09	-34,19732441	intron_variant
ENSSSCG00000009490	DCT	5,65767E-16	-34,1140357	intron_variant
ENSSSCG00000017511	PLXDC1	8,53381E-12	-34,06606607	intron_variant
ENSSSCG00000038963	PRKX	9,80725E-12	-34,01819593	intron_variant
ENSSSCG00000000951	CSRP2	1,14416E-16	-34,01607283	intron_variant
ENSSSCG00000006956	ZC3H3	3,15081E-14	-34,00955366	intron_variant
ENSSSCG00000026161	E2F6	1,42122E-10	-33,98915315	3_prime_UTR_variant
ENSSSCG00000016098	ORC2	8,64109E-08	-33,98701413	intron_variant
ENSSSCG00000001539	PPARD	2,03891E-11	-33,95602123	intron_variant
ENSSSCG00000027407	MYH14	4,60044E-18	-33,90323991	intron_variant
ENSSSCG00000021285	RNF216	1,28103E-13	-33,80213762	intron_variant
ENSSSCG00000001555	SLC26A8	4,66338E-27	-33,75	intron_variant
ENSSSCG00000016618	CPED1	5,302E-25	-33,63386721	intron_variant
ENSSSCG00000017473	TOP2A	6,89654E-21	-33,63375131	upstream_gene_variant
ENSSSCG00000023445	AVL9	6,5338E-112	-33,60886556	intron_variant
ENSSSCG00000014891	GAB2	2,55593E-36	-33,56347222	intron_variant
ENSSSCG00000016432	PRKAG2	1,68408E-17	-33,55349516	intron_variant
ENSSSCG00000008058	KCTD5	1,86136E-25	-33,53834969	intron_variant
ENSSSCG00000007022	ANK1	4,08232E-07	-33,5039052	intron_variant
ENSSSCG00000026748	PLK1	2,2345E-41	-33,46779031	intron_variant
ENSSSCG00000005465	SUSD1	1,84312E-22	-33,38267286	intron_variant
ENSSSCG00000003823	C1orf87	3,34795E-12	-33,3479021	intron_variant
ENSSSCG00000015255	IGSF9B	1,94031E-23	-33,30818177	downstream_gene_variant
ENSSSCG00000010638	TCF7L2	2,6968E-13	-33,26355662	intron_variant
ENSSSCG00000016691	JAZF1	6,83011E-11	-33,23278029	intron_variant
ENSSSCG00000033919	DCLK1	5,6798E-10	-33,09415826	intron_variant
ENSSSCG00000014337	ETF1	1,5022E-39	-33,04253941	intron_variant
ENSSSCG00000062502	-	2,44107E-20	-33,00957107	intron_variant,non_coding_transcript_variant
ENSSSCG00000038526	FBXL18	1,10588E-11	-32,99174539	intron_variant
ENSSSCG00000009125	-	3,16938E-30	-32,97591988	intron_variant
ENSSSCG00000005935	AGO2	1,83933E-08	-32,94000064	intron_variant
ENSSSCG00000012878	IGHMBP2	2,93195E-19	-32,88888889	downstream_gene_variant
ENSSSCG00000007606	TRRAP	5,19908E-21	-32,85622292	intron_variant
ENSSSCG00000034720	IQCK	4,09908E-12	-32,82809381	intron_variant
ENSSSCG00000022312	RHPN2	7,93238E-17	-32,82025095	intron_variant
ENSSSCG00000042031	-	2,57809E-37	-32,68185756	intron_variant,non_coding_transcript_variant
ENSSSCG00000058532	-	1,58192E-12	-32,64821487	upstream_gene_variant
ENSSSCG00000000577	GYS2	5,15426E-17	-32,63291855	intron_variant
ENSSSCG00000002675	DNAAF1	1,079E-07	-32,54755997	intron_variant
ENSSSCG00000024081	NRBF2	2,00577E-22	-32,52414969	intron_variant
ENSSSCG00000009779	MPHOSPH9	1,6218E-09	-32,50310759	intron_variant
ENSSSCG00000013299	APIP	2,84635E-10	-32,48752586	intron_variant

ENSSSCG00000001097	RIPOR2	5,95885E-42	-32,48549226	intron_variant
ENSSSCG00000040713	TXLNG	5,2311E-44	-32,47144956	intron_variant
ENSSSCG00000017012	SLIT3	1,56241E-09	-32,44103042	intron_variant
ENSSSCG00000049152	-	4,84963E-21	-32,40201271	upstream_gene_variant
ENSSSCG00000011117	UPF2	8,49272E-39	-32,38583411	intron_variant
ENSSSCG00000027854	HSD17B6	2,62202E-21	-32,36303926	intron_variant
ENSSSCG00000007248	ASXL1	6,24298E-26	-32,22222222	intron_variant
ENSSSCG00000036695	IGF2BP3	1,99761E-32	-32,17605059	intron_variant
ENSSSCG00000055622	-	1,55934E-11	-32,15746192	intron_variant,non_coding_transcript_variant
ENSSSCG00000032814	DNAH7	8,18049E-12	-32,13564214	upstream_gene_variant
ENSSSCG00000008512	TTC27	1,64898E-17	-32,11058131	intron_variant
ENSSSCG00000003898	MKNK1	2,57567E-08	-32,10526316	intron_variant
ENSSSCG00000016716	GSDME	1,57892E-18	-32,07662215	intron_variant
ENSSSCG00000001862	ETFA	8,36469E-42	-32,0140115	intron_variant
ENSSSCG00000059723	-	3,68115E-14	-31,99767712	intron_variant,non_coding_transcript_variant
ENSSSCG00000048250	-	1,87079E-49	-31,96662652	intron_variant,non_coding_transcript_variant
ENSSSCG00000059578	C16orf74	6,83202E-09	-31,89215448	downstream_gene_variant
ENSSSCG00000018049	PRPSAP2	2,37251E-13	-31,87041293	intron_variant
ENSSSCG00000036169	UMAD1	2,2585E-14	-31,85840708	intron_variant
ENSSSCG00000029474	AVEN	3,6402E-24	-31,80180303	intron_variant
ENSSSCG00000052507	-	5,18765E-17	-31,73278657	upstream_gene_variant
ENSSSCG00000025711	-	2,82163E-17	-31,69104657	intron_variant
ENSSSCG00000014881	CLNS1A	5,29655E-21	-31,68610817	intron_variant
ENSSSCG00000060643	-	5,18169E-15	-31,67230121	upstream_gene_variant
ENSSSCG00000028925	CEP95	1,76093E-23	-31,63962679	intron_variant
ENSSSCG00000000665	RIMKLB	7,12824E-20	-31,63391996	intron_variant
ENSSSCG00000011112	CDC123	3,66334E-54	-31,63215321	intron_variant
ENSSSCG00000025514	RUFY3	5,02958E-13	-31,61733162	intron_variant
ENSSSCG00000016008	CWC22	2,08872E-39	-31,61613426	intron_variant
ENSSSCG00000035371	CSGALNACT1	5,3658E-12	-31,57194995	intron_variant
ENSSSCG00000057567	-	5,47573E-31	-31,55419347	downstream_gene_variant
ENSSSCG00000033674	PRKCB	1,48252E-15	-31,51913876	intron_variant
ENSSSCG00000002495	SYNE3	2,09549E-26	-31,47133546	intron_variant
ENSSSCG00000062275	-	6,17883E-11	-31,46929825	intron_variant
ENSSSCG00000058289	-	2,67342E-14	-31,45961266	upstream_gene_variant
ENSSSCG00000028359	BST1	9,26247E-30	-31,37760328	downstream_gene_variant
ENSSSCG00000006475	IQGAP3	9,95598E-17	-31,35119223	intron_variant
ENSSSCG00000005740	SARDH	3,84163E-13	-31,27335912	intron_variant
ENSSSCG00000005743	VAV2	8,62286E-32	-31,26425335	intron_variant
ENSSSCG00000031628	PRPH2	1,61335E-28	-31,2261107	intron_variant
ENSSSCG00000033221	-	5,08673E-07	-31,1965812	upstream_gene_variant
ENSSSCG00000016532	WDR91	2,67603E-22	-31,17647059	intron_variant
ENSSSCG00000037174	ASMTL	1,62583E-13	-31,14917127	intron_variant
ENSSSCG00000052785	-	5,52569E-09	-31,06344489	downstream_gene_variant
ENSSSCG00000003314	NLRP11	1,66452E-19	-30,99672081	downstream_gene_variant
ENSSSCG00000010563	BTRC	5,49369E-31	-30,93579109	intron_variant

ENSSSCG00000023320	-	9,54716E-17	-30,89712416	intron_variant
ENSSSCG00000008428	MSH2	1,48002E-17	-30,87301587	intron_variant
ENSSSCG00000036837	HS3ST4	1,40816E-17	-30,80580016	intron_variant
ENSSSCG00000028723	CCDC102A	6,67075E-17	-30,71762323	intron_variant
ENSSSCG00000024158	ANO1	2,23815E-13	-30,70174495	intron_variant
ENSSSCG00000060726	-	4,59296E-10	-30,63476199	intron_variant,non_coding_transcript_variant
ENSSSCG00000032971	SMC1B	1,81644E-11	-30,62187218	intron_variant
ENSSSCG00000054550	-	2,65774E-10	-30,57960382	upstream_gene_variant
ENSSSCG00000001987	RIPK3	1,47937E-15	-30,4930569	upstream_gene_variant
ENSSSCG00000033204	-	6,28151E-07	-30,45033383	3_prime_UTR_variant
ENSSSCG00000026041	MAP3K5	4,14432E-30	-30,43521986	intron_variant
ENSSSCG00000010655	GFRA1	5,87497E-19	-30,43478261	intron_variant
ENSSSCG00000022661	FCHO2	3,28532E-08	-30,38058041	intron_variant
ENSSSCG00000044127	-	3,6214E-15	-30,37545788	intron_variant
ENSSSCG00000017918	ARRB2	6,31584E-09	-30,22269087	3_prime_UTR_variant
ENSSSCG00000022553	TNRC18	5,62193E-12	-30,21745884	intron_variant
ENSSSCG00000017534	HOXB3	1,08439E-07	-30,12675668	upstream_gene_variant
ENSSSCG00000011313	LIMD1	5,99759E-29	-30,11896552	intron_variant
ENSSSCG00000022398	NUCKS1	9,46354E-08	-30,06680865	upstream_gene_variant
ENSSSCG00000030424	USP31	2,02582E-10	-30,05783561	intron_variant
ENSSSCG00000015632	-	1,21791E-14	-30,02530126	downstream_gene_variant
ENSSSCG00000061065	-	1,7081E-07	-29,99691786	intron_variant
ENSSSCG00000058048	TTPA	1,31167E-32	-29,99097709	intron_variant
ENSSSCG00000038296	SH3RF3	2,42595E-12	-29,9768215	intron_variant
ENSSSCG00000003921	ZSWIM5	3,77234E-15	-29,96804957	intron_variant
ENSSSCG00000017172	ST6GALNAC1	2,86816E-19	-29,91728242	intron_variant
ENSSSCG00000001499	DST	1,2246E-13	-29,88317278	intron_variant
ENSSSCG00000011176	SUSD4	7,7727E-14	-29,87064677	intron_variant
ENSSSCG00000002859	ANKRD27	3,37772E-09	-29,84290574	intron_variant
ENSSSCG00000040673	TMEM140	4,16573E-13	-29,83974818	intron_variant
ENSSSCG00000004311	RNGTT	8,46598E-13	-29,81283422	intron_variant
ENSSSCG00000025243	SGIP1	5,64321E-13	-29,80840088	intron_variant
ENSSSCG00000015082	DSCAML1	6,60611E-06	-29,70787795	intron_variant
ENSSSCG00000025221	PAXIP1	7,52351E-08	-29,64709022	intron_variant
ENSSSCG00000016403	VIPR2	1,84818E-07	-29,53953511	intron_variant
ENSSSCG00000000693	NCAPD2	1,26806E-09	-29,50843017	intron_variant
ENSSSCG00000056681	-	9,83264E-20	-29,48277455	non_coding_transcript_exon_variant
ENSSSCG00000002552	TDRD9	2,76031E-09	-29,48176157	intron_variant
ENSSSCG00000026196	FAM13A	2,19175E-06	-29,48116415	intron_variant
ENSSSCG00000033175	SASH1	1,68392E-08	-29,45286381	missense_variant
ENSSSCG00000038285	KLHL36	7,12387E-06	-29,44705269	intron_variant
ENSSSCG00000001595	DAAM2	1,80712E-11	-29,41798942	intron_variant
ENSSSCG00000006730	MAN1A2	2,21516E-30	-29,41401122	intron_variant
ENSSSCG00000010824	RAB3GAP2	2,35171E-10	-29,37062937	downstream_gene_variant
ENSSSCG00000014101	AP3B1	1,09485E-10	-29,36233246	intron_variant
ENSSSCG00000036096	-	2,31503E-09	-29,35842601	downstream_gene_variant

ENSSSCG00000036002	TGIF2	1,16263E-09	-29,33127906	intron_variant
ENSSSCG00000016674	MINDY4	4,44181E-09	-29,31789737	intron_variant
ENSSSCG00000005742	DBH	2,07923E-06	-29,31061034	intron_variant
ENSSSCG00000001052	PHACTR1	4,4849E-08	-29,28691679	intron_variant
ENSSSCG00000020671	SLC18A2	1,01104E-12	-29,25670498	intron_variant
ENSSSCG00000037764	POU6F2	8,18911E-17	-29,2380325	intron_variant
ENSSSCG00000035103	PIGL	1,76848E-15	-29,23485184	intron_variant
ENSSSCG00000021769	ZNF341	8,82073E-17	-29,21393321	intron_variant
ENSSSCG00000007100	SLC24A3	3,18104E-12	-29,21052632	intron_variant
ENSSSCG00000021519	CPNE2	9,99586E-11	-29,21051433	intron_variant
ENSSSCG00000014894	TENM4	1,62016E-07	-29,20145191	intron_variant
ENSSSCG00000012848	EPS8L2	1,21401E-29	-29,18075595	intron_variant
ENSSSCG00000033689	GPS1	4,8027E-06	-29,17518746	missense_variant
ENSSSCG00000016318	ASB18	4,76748E-13	-29,17436917	intron_variant
ENSSSCG00000002872	GARRE1	7,68074E-20	-29,16359074	intron_variant
ENSSSCG00000062876	SLC25A30	9,61402E-13	-29,12732159	downstream_gene_variant
ENSSSCG00000016834	LMBRD2	1,92269E-10	-29,09455598	downstream_gene_variant
ENSSSCG00000007944	CORO7	3,39684E-16	-29,08727248	intron_variant
ENSSSCG00000007179	STK35	6,64461E-31	-29,02801601	upstream_gene_variant
ENSSSCG00000000139	IFT27	1,10759E-26	-29,02753639	intron_variant
ENSSSCG00000039647	EIF3F	1,91989E-05	-29,01785714	downstream_gene_variant
ENSSSCG00000007247	KIF3B	5,38376E-17	-28,92531198	upstream_gene_variant
ENSSSCG00000000137	NCF4	1,25113E-45	-28,91527236	downstream_gene_variant
ENSSSCG00000015246	ST14	1,58086E-38	-28,88855907	intron_variant
ENSSSCG00000002664	GSE1	5,20721E-27	-28,86474934	intron_variant
ENSSSCG00000044139	-	2,34451E-83	-28,84868754	intron_variant,non_coding_transcript_variant
ENSSSCG00000053358	RAB40B	1,30021E-40	-28,82191758	intron_variant
ENSSSCG00000038949	NALCN	7,76445E-11	-28,79319193	intron_variant
ENSSSCG00000016237	DOCK10	9,18658E-10	-28,72829351	intron_variant
ENSSSCG00000036178	CCDC167	2,90973E-54	-28,72458387	upstream_gene_variant
ENSSSCG00000026409	TYR	1,3948E-16	-28,70551671	intron_variant
ENSSSCG00000060583	-	2,39379E-10	-28,70239187	downstream_gene_variant
ENSSSCG00000004561	HERC1	3,60871E-24	-28,69343845	intron_variant
ENSSSCG00000009142	SEC24B	7,37091E-17	-28,69276966	intron_variant
ENSSSCG00000002243	SLC12A6	1,09973E-20	-28,58731151	intron_variant
ENSSSCG00000016442	AOC1	6,78664E-10	-28,58516649	upstream_gene_variant
ENSSSCG00000026996	-	8,10743E-23	-28,58249564	intron_variant
ENSSSCG00000005291	TLE1	1,40875E-26	-28,57966841	intron_variant
ENSSSCG00000000682	GNB3	1,39698E-22	-28,57142857	intron_variant
ENSSSCG00000038811	MOB3B	3,38206E-06	-28,53048463	intron_variant
ENSSSCG00000023785	TMEM156	5,28204E-12	-28,50126112	downstream_gene_variant
ENSSSCG00000027349	TBC1D14	3,47251E-21	-28,45692453	intron_variant
ENSSSCG00000008018	MAPK8IP3	1,56889E-17	-28,43396116	intron_variant
ENSSSCG00000032659	ASMT	2,59876E-09	-28,42485109	upstream_gene_variant
ENSSSCG00000046928	-	4,5867E-46	-28,36564192	intron_variant,non_coding_transcript_variant
ENSSSCG00000002718	FA2H	1,20519E-18	-28,36120401	intron_variant

ENSSSCG00000031776	-	2,79172E-06	-28,32822086	intron_variant
ENSSSCG00000040207	P2RY2	7,32173E-06	-28,30410607	intron_variant
ENSSSCG00000037645	COTL1	4,30068E-06	-28,3033427	intron_variant
ENSSSCG00000036208	SHMT1	2,66207E-15	-28,28128967	intron_variant
ENSSSCG00000033544	NTF3	2,63425E-05	-28,26560232	intron_variant
ENSSSCG00000009230	WDFY3	1,44559E-41	-28,24352794	intron_variant
ENSSSCG00000008803	ATP8A1	3,06061E-07	-28,23848238	upstream_gene_variant
ENSSSCG00000057105	PRPS2	2,14544E-08	-28,23727221	intron_variant
ENSSSCG00000017783	MYO18A	2,72885E-08	-28,2037769	intron_variant
ENSSSCG00000046151	-	2,52872E-14	-28,17267121	intron_variant,non_coding_transcript_variant
ENSSSCG00000014088	IQGAP2	1,02297E-08	-28,1598979	intron_variant
ENSSSCG00000007955	CLUAP1	2,7248E-08	-28,15610267	intron_variant
ENSSSCG00000008986	SEPTIN11	5,66573E-17	-28,13444109	intron_variant
ENSSSCG00000022190	UBE2V2	1,45544E-08	-28,0955374	intron_variant
ENSSSCG00000060130	-	6,46104E-07	-28,06441012	non_coding_transcript_exon_variant
ENSSSCG00000040107	GMEB2	5,25316E-09	-28,03484321	intron_variant
ENSSSCG00000055574	-	3,46723E-28	-28,01446945	intron_variant,non_coding_transcript_variant
ENSSSCG00000023235	MAN1C1	2,10269E-08	-28,00556642	intron_variant
ENSSSCG00000003405	PEX14	1,81976E-07	-27,99555757	intron_variant
ENSSSCG00000007501	BMP7	5,22134E-07	-27,97959184	intron_variant
ENSSSCG00000009543	-	3,19366E-05	-27,94117647	intron_variant
ENSSSCG00000042093	-	6,7613E-17	-27,93528412	downstream_gene_variant
ENSSSCG00000031946	GPC5	5,75289E-44	-27,93021134	intron_variant
ENSSSCG00000062261	U6	0,000541167	-27,90273556	upstream_gene_variant
ENSSSCG00000063182	-	7,96254E-15	-27,85931638	downstream_gene_variant
ENSSSCG00000063378	STPG4	1,14047E-24	-27,82789103	downstream_gene_variant
ENSSSCG00000053428	-	1,00703E-59	-27,65435121	downstream_gene_variant
ENSSSCG00000008563	KCNK3	5,91995E-08	-27,65257698	intron_variant
ENSSSCG00000016570	TSPAN33	2,76175E-12	-27,64400426	upstream_gene_variant
ENSSSCG00000011104	CUL2	1,30103E-10	-27,63758353	downstream_gene_variant
ENSSSCG00000008043	TRAF7	1,65822E-11	-27,63254039	intron_variant
ENSSSCG00000009019	RPS3A	2,58371E-06	-27,62237762	intron_variant
ENSSSCG00000054203	-	9,61497E-13	-27,57355171	upstream_gene_variant
ENSSSCG00000037318	TRABD2B	2,72874E-10	-27,56756757	intron_variant
ENSSSCG00000000486	NUP107	1,6755E-14	-27,51644503	intron_variant
ENSSSCG00000011101	ITGB1	1,70669E-07	-27,49423847	intron_variant
ENSSSCG00000061951	-	1,61314E-10	-27,48702743	intron_variant,non_coding_transcript_variant
ENSSSCG00000021490	PRDM16	1,97083E-08	-27,45150146	intron_variant
ENSSSCG00000027568	BLK	5,51295E-11	-27,4377805	intron_variant
ENSSSCG00000049255	-	3,79037E-16	-27,4305444	intron_variant,non_coding_transcript_variant
ENSSSCG00000016334	SCLY	5,0936E-11	-27,39798157	intron_variant
ENSSSCG00000054246	-	3,37614E-12	-27,37894144	downstream_gene_variant
ENSSSCG00000023992	CEP112	1,90203E-09	-27,35868093	intron_variant
ENSSSCG00000007915	TMEM114	5,7236E-10	-27,33031674	intron_variant
ENSSSCG00000013276	PRDM11	4,39687E-05	-27,32854148	intron_variant
ENSSSCG00000006888	SLC44A3	3,96098E-05	-27,31712732	intron_variant

ENSSSCG00000022168	APBB2	2,06607E-37	-27,3164016	intron_variant
ENSSSCG00000002708	TMEM231	1,7689E-16	-27,3046398	intron_variant
ENSSSCG00000060437	-	5,51004E-08	-27,2955875	non_coding_transcript_exon_variant
ENSSSCG00000009290	MIPEP	1,17654E-30	-27,29044834	intron_variant
ENSSSCG00000040639	THAP12	1,18469E-44	-27,20781226	intron_variant
ENSSSCG00000015885	PLA2R1	1,03014E-57	-27,20303041	intron_variant
ENSSSCG00000051558	SYCP2L	2,41054E-22	-27,15927395	intron_variant
ENSSSCG00000044219	-	4,90905E-07	-27,14187665	upstream_gene_variant
ENSSSCG00000056189	-	5,86941E-07	-27,13785287	non_coding_transcript_exon_variant
ENSSSCG00000035243	RAB27B	1,95805E-09	-27,05170924	intron_variant
ENSSSCG00000005699	HMCN2	1,94448E-05	-26,96585366	intron_variant
ENSSSCG00000013457	DOT1L	1,90634E-18	-26,88651935	intron_variant
ENSSSCG00000009495	-	6,19814E-17	-26,88645545	intron_variant
ENSSSCG00000018027	-	7,9895E-08	-26,88179636	downstream_gene_variant
ENSSSCG00000032967	CACNB3	1,54341E-06	-26,85618729	upstream_gene_variant
ENSSSCG00000007500	-	4,71968E-05	-26,816369	downstream_gene_variant
ENSSSCG00000009957	MYO18B	1,35515E-06	-26,81126203	intron_variant
ENSSSCG00000009270	IFT88	2,91061E-33	-26,78373561	intron_variant
ENSSSCG00000025629	COLEC11	2,05832E-08	-26,7674468	downstream_gene_variant
ENSSSCG00000006037	OXR1	3,00777E-56	-26,74016135	intron_variant
ENSSSCG00000000396	STAT2	1,79711E-25	-26,73312603	downstream_gene_variant
ENSSSCG00000056614	-	1,01208E-10	-26,71960739	non_coding_transcript_exon_variant
ENSSSCG00000035196	BTBD8	3,40793E-22	-26,69360827	intron_variant
ENSSSCG00000015866	FMNL2	1,04991E-55	-26,69020182	intron_variant
ENSSSCG00000002037	CDH24	6,74425E-34	-26,65765004	intron_variant
ENSSSCG00000040538	WDFY2	6,26248E-16	-26,58063121	intron_variant
ENSSSCG00000029507	RASGEF1B	2,07061E-16	-26,55587006	downstream_gene_variant
ENSSSCG00000001391	CCHCR1	2,61457E-36	-26,54523943	upstream_gene_variant
ENSSSCG00000031988	MYOCD	0,000239521	-26,52628604	intron_variant
ENSSSCG00000011552	TTLL3	6,7883E-11	-26,5164107	intron_variant
ENSSSCG00000039056	GAS7	9,04753E-20	-26,50201397	intron_variant
ENSSSCG00000023710	REEP1	5,01814E-16	-26,46987425	intron_variant
ENSSSCG00000006767	MAGI3	4,79253E-09	-26,45903571	intron_variant
ENSSSCG00000024754	BBS9	4,63751E-10	-26,43486544	intron_variant
ENSSSCG00000023357	-	1,63456E-07	-26,42439974	upstream_gene_variant
ENSSSCG00000014136	VCAN	3,79262E-20	-26,4074635	intron_variant
ENSSSCG00000003396	CLSTN1	1,80499E-07	-26,36554622	intron_variant
ENSSSCG00000017104	NSUN2	5,6353E-06	-26,36363636	intron_variant
ENSSSCG00000016845	NUP155	6,20011E-24	-26,35498896	intron_variant
ENSSSCG00000023907	AFAP1	4,20862E-07	-26,34408602	intron_variant
ENSSSCG00000023669	SNORD30	1,33227E-06	-26,34016237	upstream_gene_variant
ENSSSCG00000037539	SORCS2	7,8486E-08	-26,30397237	intron_variant
ENSSSCG00000031782	ARL3	5,87479E-10	-26,2847181	intron_variant
ENSSSCG00000059490	-	3,79486E-10	-26,28148475	upstream_gene_variant
ENSSSCG00000013110	TMEM109	4,37654E-17	-26,22553854	intron_variant
ENSSSCG00000008183	MRPL30	7,19711E-20	-26,22257278	intron_variant

ENSSSCG00000027846	RRP15	2,35566E-07	-26,21096631	intron_variant
ENSSSCG00000048633	-	3,05729E-22	-26,16902825	non_coding_transcript_exon_variant
ENSSSCG00000016824	RAI14	3,33411E-07	-26,15384615	intron_variant
ENSSSCG00000034776	TAFA5	9,69487E-09	-26,11084016	intron_variant
ENSSSCG00000012072	DSCAM	2,8885E-08	-26,10488246	intron_variant
ENSSSCG00000061647	CASQ2	6,26717E-08	-26,10011001	3_prime_UTR_variant
ENSSSCG00000017213	JPT1	1,29077E-07	-26,0925302	intron_variant
ENSSSCG00000057278	-	0,000133506	-26,08678344	intron_variant,non_coding_transcript_variant
ENSSSCG00000009012	TMEM154	4,14685E-05	-26,07931727	upstream_gene_variant
ENSSSCG00000055213	GNA12	7,69708E-11	-26,07207716	intron_variant
ENSSSCG00000013388	PDE3B	1,22452E-10	-26,04835314	intron_variant
ENSSSCG00000016412	LMBR1	5,45609E-32	-26,02365691	intron_variant
ENSSSCG00000027115	-	4,45132E-08	-26,02341877	intron_variant
ENSSSCG00000016970	-	2,92555E-06	-26,01398601	intron_variant
ENSSSCG00000004018	AFDN	2,61972E-13	-26,00872144	intron_variant
ENSSSCG00000036990	DIO3	2,11178E-15	-25,99422216	intron_variant
ENSSSCG00000058255	-	7,05243E-07	-25,97114708	intron_variant
ENSSSCG00000010052	BCR	2,60318E-10	-25,9088463	intron_variant
ENSSSCG00000039952	ZFAND3	1,247E-05	-25,89481774	intron_variant
ENSSSCG00000022618	CMTM8	1,49821E-05	-25,89473684	intron_variant
ENSSSCG00000014399	ARHGAP26	4,06709E-20	-25,89401893	intron_variant
ENSSSCG00000009568	ZMYM5	8,32994E-86	-25,88431944	upstream_gene_variant
ENSSSCG00000035357	BCL2L1	1,57998E-06	-25,86206897	intron_variant
ENSSSCG00000010894	TP53BP2	2,10456E-07	-25,85110363	intron_variant
ENSSSCG00000005751	COL5A1	4,73705E-11	-25,84616109	intron_variant
ENSSSCG00000000002	-	5,82452E-19	-25,84198331	intron_variant
ENSSSCG00000054314	-	2,17197E-08	-25,83471302	upstream_gene_variant
ENSSSCG00000011872	SEMA5B	1,07513E-22	-25,79769068	intron_variant
ENSSSCG00000007010	ZMAT4	1,37307E-28	-25,74078535	intron_variant
ENSSSCG00000023151	GRIN2C	1,0337E-49	-25,73384571	intron_variant
ENSSSCG00000008499	STRN	7,04619E-08	-25,72776809	intron_variant
ENSSSCG00000058470	MYL12B	5,67087E-13	-25,70654734	downstream_gene_variant
ENSSSCG00000008991	FRAS1	7,63321E-58	-25,67748624	intron_variant
ENSSSCG00000014813	PHOX2A	1,06289E-11	-25,66609791	intron_variant
ENSSSCG00000007917	RBFOX1	6,47727E-08	-25,63230241	intron_variant
ENSSSCG00000009298	CDK8	0,000108384	-25,60256633	downstream_gene_variant
ENSSSCG00000053473	-	3,74828E-09	-25,59656287	intron_variant
ENSSSCG00000030947	ZFAT	2,42048E-22	-25,52358761	upstream_gene_variant
ENSSSCG00000021132	PLD5	4,66856E-08	-25,52146465	intron_variant
ENSSSCG00000033452	-	8,36178E-19	-25,51662174	intron_variant
ENSSSCG00000037298	-	9,06682E-07	-25,45274635	upstream_gene_variant
ENSSSCG00000055832	AFF3	2,31467E-45	-25,44505789	intron_variant
ENSSSCG00000012435	MAGT1	7,59764E-24	-25,44416688	intron_variant
ENSSSCG00000033809	IFITM10	2,72308E-07	-25,4257975	intron_variant
ENSSSCG00000040162	NUPR1	8,00381E-14	-25,4210091	upstream_gene_variant
ENSSSCG00000026579	LEKR1	8,04425E-06	-25,41191794	intron_variant

ENSSSCG00000025203	KNOP1	1,41596E-34	-25,40777335	missense_variant
ENSSSCG00000036549	DPYSL3	5,7551E-10	-25,38426865	intron_variant
ENSSSCG00000011616	COPG1	1,44013E-08	-25,36644589	intron_variant
ENSSSCG00000002516	WARS1	1,34021E-22	-25,31599613	upstream_gene_variant
ENSSSCG00000009896	BICDL1	6,65626E-09	-25,30959752	intron_variant
ENSSSCG00000012021	MRPL39	2,63412E-13	-25,28033228	intron_variant
ENSSSCG00000008730	DRD5	1,48545E-06	-25,25044723	upstream_gene_variant
ENSSSCG00000000748	IQSEC3	4,25893E-10	-25,25020058	intron_variant
ENSSSCG00000008464	MTA3	4,92964E-06	-25,2415198	intron_variant
ENSSSCG00000012817	FUNDC2	1,14813E-14	-25,23741491	3_prime_UTR_variant
ENSSSCG00000007264	CDK5RAP1	1,2718E-14	-25,23548637	upstream_gene_variant
ENSSSCG00000030095	ZBTB16	2,45213E-06	-25,22605083	intron_variant
ENSSSCG00000009754	TMEM132B	3,16265E-50	-25,20380958	intron_variant
ENSSSCG00000004843	TRPM1	6,45563E-06	-25,19721794	intron_variant
ENSSSCG00000013363	LDHC	2,16221E-05	-25,18939394	intron_variant
ENSSSCG00000003433	PLOD1	3,95962E-08	-25,18204948	intron_variant
ENSSSCG00000023001	CCDC149	2,936E-22	-25,17424191	upstream_gene_variant
ENSSSCG00000013316	WT1	1,10681E-13	-25,16516517	intron_variant
ENSSSCG00000023793	NUP42	1,31324E-07	-25,15273502	intron_variant
ENSSSCG00000029541	PSD2	1,19263E-10	-25,12195122	intron_variant
ENSSSCG00000014203	MCC	9,43921E-06	-25,12154827	intron_variant
ENSSSCG00000013298	PDHX	5,20702E-08	-25,1188345	intron_variant
ENSSSCG00000015446	PNPLA8	1,10608E-38	-25,11823919	intron_variant
ENSSSCG00000010971	ARID3C	6,07611E-22	-25,11635423	upstream_gene_variant
ENSSSCG00000007575	RADIL	4,78464E-13	-25,09787938	intron_variant
ENSSSCG00000010664	ENO4	8,62284E-12	-25,09340481	intron_variant
ENSSSCG00000001234	TRIM10	8,23911E-15	-25,08074396	upstream_gene_variant
ENSSSCG00000033272	DNAJB6	1,11708E-11	-25,06583992	intron_variant
ENSSSCG00000040650	KCNH1	0,000416434	-25,04504505	intron_variant
ENSSSCG00000040288	ARNT2	1,99236E-08	-25,04325304	intron_variant
ENSSSCG00000053446	-	1,59094E-81	-25,03680018	non_coding_transcript_exon_variant
ENSSSCG00000026110	SRPK2	2,61229E-23	25,00516011	intron_variant
ENSSSCG00000011416	DOCK3	1,6139E-07	25,00537288	3_prime_UTR_variant
ENSSSCG00000043116	-	6,63007E-10	25,0070028	downstream_gene_variant
ENSSSCG00000003712	OSBPL1A	5,06195E-07	25,00842602	intron_variant
ENSSSCG00000031423	UPK3A	5,32369E-07	25,01906941	intron_variant
ENSSSCG00000002464	PRIMA1	0,00030521	25,02422872	intron_variant
ENSSSCG00000017732	ZNF207	3,29028E-12	25,03275681	intron_variant
ENSSSCG00000016763	GLI3	3,72563E-06	25,04860423	intron_variant
ENSSSCG00000061230	-	3,4806E-13	25,06067664	upstream_gene_variant
ENSSSCG00000034844	TBCE	7,49923E-07	25,06865349	intron_variant
ENSSSCG00000009432	DGKD	2,96354E-44	25,06998679	intron_variant
ENSSSCG00000003377	ACOT7	2,82825E-05	25,07260651	intron_variant
ENSSSCG00000016510	UBN2	1,86629E-25	25,0950899	intron_variant
ENSSSCG00000009431	DGKH	1,70413E-07	25,108857	intron_variant
ENSSSCG00000017265	AXIN2	3,75398E-05	25,12388347	intron_variant

ENSSSCG00000015430	RINT1	2,74555E-12	25,12825296	intron_variant
ENSSSCG00000039751	NLRC5	6,60819E-06	25,14103862	intron_variant
ENSSSCG00000014794	NUP98	3,09104E-12	25,14335369	intron_variant
ENSSSCG00000001049	HIVEP1	1,2662E-10	25,16845613	intron_variant
ENSSSCG00000034767	LAMA1	1,13168E-07	25,2	intron_variant
ENSSSCG00000063417	-	1,26518E-08	25,20748277	intron_variant
ENSSSCG00000005587	NEK6	1,91298E-08	25,21748913	intron_variant
ENSSSCG00000035421	ATP8A2	1,61107E-17	25,22881278	intron_variant
ENSSSCG00000031738	COL24A1	4,54775E-73	25,23540732	intron_variant
ENSSSCG00000035929	-	0,00011572	25,23825967	intron_variant
ENSSSCG00000052178	-	3,35205E-82	25,25083886	synonymous_variant
ENSSSCG00000025355	ZNF146	4,45288E-18	25,2524292	intron_variant
ENSSSCG00000034763	IRS2	1,22432E-08	25,26359833	intron_variant
ENSSSCG00000002968	-	8,33991E-10	25,26840548	upstream_gene_variant
ENSSSCG00000049777	-	2,4645E-20	25,26841708	upstream_gene_variant
ENSSSCG00000036716	RPL37A	2,50178E-08	25,2718083	downstream_gene_variant
ENSSSCG00000023125	CNTNAP4	7,57071E-05	25,27557467	intron_variant
ENSSSCG00000035854	RAB11FIP3	2,37792E-09	25,27654386	intron_variant
ENSSSCG00000017126	NARF	2,33284E-18	25,279334	intron_variant
ENSSSCG00000021562	PLXNA4	0,000164088	25,28813559	intron_variant
ENSSSCG00000011121	-	2,24121E-05	25,29158993	intron_variant
ENSSSCG00000001680	ABCC10	1,72776E-08	25,2980253	upstream_gene_variant
ENSSSCG00000017735	RHOT1	3,83341E-20	25,29894172	intron_variant
ENSSSCG00000011183	ATP2C1	2,28104E-17	25,30462185	intron_variant
ENSSSCG00000025357	LDAH	3,02314E-13	25,30669345	intron_variant
ENSSSCG00000031174	FBP1	2,18795E-11	25,30925808	upstream_gene_variant
ENSSSCG00000022638	ATP12A	5,90742E-10	25,31601473	intron_variant
ENSSSCG00000024083	U6	2,05603E-05	25,32237989	downstream_gene_variant
ENSSSCG00000033093	RGS6	2,25298E-12	25,32394522	intron_variant
ENSSSCG00000008898	-	0,000176182	25,33815965	upstream_gene_variant
ENSSSCG00000004454	ME1	3,12408E-11	25,33826196	downstream_gene_variant
ENSSSCG00000059435	RNF24	4,63468E-20	25,34126386	intron_variant
ENSSSCG00000000020	PHF21B	5,02287E-14	25,3530683	intron_variant
ENSSSCG00000053371	NLRP2B	6,95726E-06	25,35457939	downstream_gene_variant
ENSSSCG00000062649	-	1,30071E-32	25,36645569	non_coding_transcript_exon_variant
ENSSSCG00000031717	ADCY8	3,24778E-12	25,37450563	intron_variant
ENSSSCG00000003640	GRIK3	0,000774942	25,42024014	intron_variant
ENSSSCG00000047974	-	5,48428E-07	25,42128069	intron_variant,non_coding_transcript_variant
ENSSSCG00000015607	HHAT	1,34693E-06	25,42136003	intron_variant
ENSSSCG00000056837	-	2,735E-08	25,42903099	intron_variant,non_coding_transcript_variant
ENSSSCG00000038231	ABCD4	1,08698E-05	25,43859649	intron_variant
ENSSSCG00000026229	-	7,60365E-05	25,44281894	intron_variant
ENSSSCG00000009768	TCTN2	2,58688E-20	25,45235251	intron_variant
ENSSSCG00000059747	NKAIN3	7,05335E-32	25,4528991	intron_variant
ENSSSCG00000014252	PRRC1	8,31327E-10	25,49450549	missense_variant
ENSSSCG00000016125	INO80D	2,13998E-11	25,50279394	intron_variant

ENSSSCG00000014599	SYT9	2,45563E-48	25,52431437	intron_variant
ENSSSCG00000014831	PAAF1	3,90623E-22	25,5647196	intron_variant
ENSSSCG00000004383	OSTM1	9,37443E-08	25,57289063	intron_variant
ENSSSCG00000009021	LRBA	8,13003E-14	25,57855915	intron_variant
ENSSSCG00000057883	-	4,48852E-06	25,61408701	upstream_gene_variant
ENSSSCG00000006083	LAPTM4B	1,40821E-05	25,62491174	intron_variant
ENSSSCG00000032842	GIPC1	6,3384E-09	25,63201923	intron_variant
ENSSSCG00000054417	-	2,63895E-12	25,63492063	downstream_gene_variant
ENSSSCG00000008697	HTT	5,44677E-06	25,65864834	intron_variant
ENSSSCG00000040732	WDR81	2,23185E-08	25,67655425	missense_variant
ENSSSCG00000009605	GFRA2	8,62242E-11	25,6765833	intron_variant
ENSSSCG00000027257	PSMB1	1,21325E-05	25,68206705	downstream_gene_variant
ENSSSCG00000010532	LOXL4	0,000681653	25,69444444	intron_variant
ENSSSCG00000004209	PTPRK	2,64839E-06	25,72807882	intron_variant
ENSSSCG00000010865	EXO1	0,000147041	25,73602794	3_prime_UTR_variant
ENSSSCG00000036056	DNAH17	1,98747E-28	25,74283379	intron_variant
ENSSSCG00000013391	PSMA1	4,90234E-11	25,74738841	intron_variant
ENSSSCG00000035318	NUP58	3,33899E-09	25,75356502	intron_variant
ENSSSCG00000015543	CACNA1E	4,10641E-24	25,79113924	intron_variant
ENSSSCG00000025483	GREB1	1,14873E-14	25,80408463	upstream_gene_variant
ENSSSCG00000038505	MSI2	3,00949E-09	25,8059368	intron_variant
ENSSSCG00000040944	SKI	1,88701E-14	25,82021087	intron_variant
ENSSSCG00000008723	HTRA3	6,84106E-06	25,82180247	intron_variant
ENSSSCG00000017514	KPNB1	8,48965E-29	25,83390479	upstream_gene_variant
ENSSSCG00000049143	-	1,82719E-05	25,84190004	downstream_gene_variant
ENSSSCG00000052379	-	8,42246E-15	25,91233028	upstream_gene_variant
ENSSSCG00000039259	-	3,28523E-17	25,92331831	intron_variant
ENSSSCG00000052201	-	1,77174E-06	25,93023256	downstream_gene_variant
ENSSSCG00000016317	AGAP1	9,85365E-11	25,93838343	intron_variant
ENSSSCG00000040403	NANOG	2,17287E-06	25,94299576	upstream_gene_variant
ENSSSCG00000040746	LRP2	3,00428E-06	25,96232293	downstream_gene_variant
ENSSSCG00000010540	ENTPD7	1,7096E-11	25,96320346	intron_variant
ENSSSCG00000005106	NTRK3	3,6701E-06	25,9963279	intron_variant
ENSSSCG00000032643	SLC12A3	3,56999E-09	26,004851	intron_variant
ENSSSCG00000016141	PLEKHM3	1,66593E-09	26,00529101	downstream_gene_variant
ENSSSCG00000000531	BICD1	9,14414E-06	26,00744879	intron_variant
ENSSSCG00000039317	SLC25A21	9,35515E-13	26,00987393	intron_variant
ENSSSCG00000012102	-	5,62856E-11	26,03530656	intron_variant
ENSSSCG00000017747	RAB11FIP4	1,58929E-05	26,04907975	3_prime_UTR_variant
ENSSSCG00000010745	FANK1	1,35063E-19	26,05641886	intron_variant
ENSSSCG00000033518	LLGL2	3,45674E-14	26,06534347	intron_variant
ENSSSCG00000017737	RNF135	1,80706E-34	26,07670108	downstream_gene_variant
ENSSSCG00000028942	NRXN3	4,37145E-12	26,08187715	intron_variant
ENSSSCG00000005466	PTBP3	1,77685E-05	26,08376429	intron_variant
ENSSSCG00000025272	HEMK1	5,09701E-05	26,10005379	upstream_gene_variant
ENSSSCG00000024562	CCR6	1,72246E-09	26,10598307	downstream_gene_variant

ENSSSCG00000017188	ZACN	1,5311E-16	26,10714766	downstream_gene_variant
ENSSSCG00000039316	GSG1	1,39629E-05	26,11528822	intron_variant
ENSSSCG00000010509	PIK3AP1	2,61154E-12	26,13082226	intron_variant
ENSSSCG00000004289	CEP162	1,13799E-06	26,13950498	downstream_gene_variant
ENSSSCG00000044128	-	0,001548876	26,14397719	intron_variant,non_coding_transcript_variant
ENSSSCG00000024166	SLC2A6	9,30406E-08	26,16506969	intron_variant
ENSSSCG00000006738	-	5,77296E-09	26,1929782	intron_variant
ENSSSCG00000035537	RUNX1	1,13717E-05	26,19986851	intron_variant
ENSSSCG00000007451	SLC2A10	1,36297E-32	26,20764366	downstream_gene_variant
ENSSSCG00000011353	PFKFB4	9,8538E-31	26,21717995	intron_variant
ENSSSCG00000016321	IQCA1	1,65615E-09	26,21819567	intron_variant
ENSSSCG00000007839	EEF2K	1,7859E-07	26,22081309	intron_variant
ENSSSCG00000037558	YY1	3,54057E-06	26,23446296	intron_variant
ENSSSCG00000021793	SMIM14	1,54064E-07	26,27855564	intron_variant
ENSSSCG00000057323	-	1,85535E-08	26,28026412	upstream_gene_variant
ENSSSCG00000062960	-	2,79738E-06	26,29250116	intron_variant
ENSSSCG00000016475	-	3,40232E-31	26,2974266	intron_variant
ENSSSCG00000019423	U4	2,07268E-06	26,2987013	upstream_gene_variant
ENSSSCG00000016259	FBXO36	3,76933E-09	26,30290815	intron_variant
ENSSSCG00000023374	SRGN	7,2132E-09	26,31472449	intron_variant
ENSSSCG00000043420	-	6,36777E-10	26,32382635	non_coding_transcript_exon_variant
ENSSSCG00000045188	U2	1,39609E-07	26,3681592	downstream_gene_variant
ENSSSCG00000023654	BRDT	2,31464E-13	26,38135494	intron_variant
ENSSSCG00000004962	CORO2B	1,14603E-09	26,39309412	intron_variant
ENSSSCG00000029331	PALLD	4,31211E-09	26,4021164	intron_variant
ENSSSCG00000052830	-	1,91513E-06	26,44499091	downstream_gene_variant
ENSSSCG00000031838	-	2,47574E-27	26,44683715	upstream_gene_variant
ENSSSCG00000017874	ATP2A3	2,07139E-11	26,4573991	intron_variant
ENSSSCG00000016256	SPHKAP	1,03015E-15	26,47507331	intron_variant
ENSSSCG00000011355	-	1,56102E-07	26,49574013	intron_variant
ENSSSCG00000053730	-	3,19886E-13	26,49854711	non_coding_transcript_exon_variant
ENSSSCG00000005975	MTSS1	2,37191E-16	26,50902057	intron_variant
ENSSSCG00000020988	-	4,32818E-23	26,52030948	intron_variant
ENSSSCG00000017109	ADAMTS16	4,1207E-06	26,53145695	intron_variant
ENSSSCG00000044784	-	8,92554E-08	26,53522948	downstream_gene_variant
ENSSSCG00000023434	PPM1L	4,14169E-17	26,5395993	intron_variant
ENSSSCG00000016448	KCNH2	8,65307E-10	26,55354449	intron_variant
ENSSSCG00000001219	TRIM27	9,28843E-35	26,55889294	intron_variant
ENSSSCG00000030361	PRKCZ	2,66254E-18	26,58075929	intron_variant
ENSSSCG00000054506	VEGFB	6,09272E-12	26,58145413	upstream_gene_variant
ENSSSCG00000002654	FBXO31	3,46168E-25	26,62314904	intron_variant
ENSSSCG00000031450	MLYCD	7,45765E-10	26,63594214	3_prime_UTR_variant
ENSSSCG00000029334	MYT1L	3,15872E-08	26,64323	intron_variant
ENSSSCG00000037056	PPP1R37	1,02023E-10	26,65377176	intron_variant
ENSSSCG00000008289	MTHFD2	2,78355E-14	26,66220305	intron_variant
ENSSSCG00000017890	PITPNM3	7,06706E-13	26,66382066	intron_variant

ENSSSCG00000014430	ABLIM3	1,21462E-11	26,67640258	intron_variant
ENSSSCG00000017551	FAM117A	8,93904E-07	26,69685852	intron_variant
ENSSSCG00000010799	COG7	2,40689E-10	26,7701533	intron_variant
ENSSSCG00000028983	TBC1D1	3,08913E-07	26,85185185	intron_variant
ENSSSCG00000038766	MAD1L1	3,74644E-25	26,85434768	intron_variant
ENSSSCG00000024665	TRAPPC12	1,01115E-20	26,85475444	intron_variant
ENSSSCG00000031700	SLC2A9	2,45983E-18	26,86314791	upstream_gene_variant
ENSSSCG00000014433	PCYOX1L	1,19602E-77	26,91051941	intron_variant
ENSSSCG00000013890	SLC5A5	2,49302E-10	26,92484324	intron_variant
ENSSSCG00000002670	USP10	1,04146E-06	26,95702897	downstream_gene_variant
ENSSSCG00000009018	SH3D19	1,58165E-07	26,96354132	missense_variant
ENSSSCG00000057024	PPP3CC	5,59204E-12	26,97027014	intron_variant
ENSSSCG00000010522	ANKRD2	2,6064E-15	26,98076923	downstream_gene_variant
ENSSSCG00000009276	XPO4	3,79671E-11	26,98207397	intron_variant
ENSSSCG00000034059	ST3GAL3	1,12461E-08	26,99642857	intron_variant
ENSSSCG00000029482	CHST15	7,38711E-15	27,00268766	intron_variant
ENSSSCG00000011646	KY	5,58797E-13	27,00328293	upstream_gene_variant
ENSSSCG00000030960	-	4,23893E-42	27,01310732	downstream_gene_variant
ENSSSCG00000011782	ABCC5	1,21853E-13	27,01662944	intron_variant
ENSSSCG00000031361	CELSR1	9,95043E-09	27,03263535	intron_variant
ENSSSCG00000007505	CTCFL	6,0865E-17	27,03807228	intron_variant
ENSSSCG00000007817	IL4R	4,32265E-07	27,09270149	intron_variant
ENSSSCG00000010302	USP54	2,06488E-26	27,09666591	intron_variant
ENSSSCG00000009185	ADH5	1,45649E-27	27,09741412	intron_variant
ENSSSCG00000027334	EIF1AD	3,8157E-11	27,1249595	3_prime_UTR_variant
ENSSSCG00000038943	TOM1	5,52839E-24	27,17838132	intron_variant
ENSSSCG00000018053	MED9	7,71885E-10	27,18365747	intron_variant
ENSSSCG00000020474	U1	4,72543E-11	27,1856496	downstream_gene_variant
ENSSSCG00000010507	TLL2	0,000207013	27,19122508	intron_variant
ENSSSCG00000029380	ZFP2	1,24972E-16	27,2184373	intron_variant
ENSSSCG00000003336	TTLL10	2,43379E-05	27,22138554	intron_variant
ENSSSCG00000043011	-	7,19868E-09	27,2448024	intron_variant,non_coding_transcript_variant
ENSSSCG00000028755	EIPR1	2,42755E-18	27,25317693	intron_variant
ENSSSCG00000042361	-	2,02007E-06	27,25695665	intron_variant,non_coding_transcript_variant
ENSSSCG00000035230	PIEZO1	6,12953E-18	27,2569957	intron_variant
ENSSSCG00000015930	DHRS9	4,34764E-08	27,26888983	intron_variant
ENSSSCG00000024077	-	5,77432E-08	27,30087551	downstream_gene_variant
ENSSSCG00000012774	DUSP9	3,64192E-08	27,33109994	downstream_gene_variant
ENSSSCG00000025784	CDH4	5,26336E-07	27,3744824	intron_variant
ENSSSCG00000023747	TTC19	8,12074E-39	27,38095238	upstream_gene_variant
ENSSSCG00000003512	EIF4G3	4,87856E-20	27,39310542	intron_variant
ENSSSCG00000027331	COL6A3	5,2382E-08	27,39812906	intron_variant
ENSSSCG00000007344	KIAA1755	1,07534E-28	27,43173006	upstream_gene_variant
ENSSSCG00000054294	-	3,40243E-13	27,4502416	intron_variant,non_coding_transcript_variant
ENSSSCG00000040275	HEBP1	1,03857E-09	27,4639978	upstream_gene_variant
ENSSSCG00000008498	HEATR5B	2,12981E-18	27,46580355	intron_variant

ENSSSCG00000008721	ABLIM2	7,56018E-06	27,53790664	intron_variant
ENSSSCG00000022451	WDR73	2,13629E-15	27,55025503	downstream_gene_variant
ENSSSCG00000000955	TBC1D22A	6,57108E-08	27,5635258	intron_variant
ENSSSCG00000017192	-	1,7441E-15	27,5764037	missense_variant
ENSSSCG00000007950	ADCY9	9,44781E-63	27,58901569	intron_variant
ENSSSCG00000003561	ZDHHC18	1,0334E-10	27,61700274	upstream_gene_variant
ENSSSCG00000029783	MKX	8,2154E-12	27,6242999	intron_variant
ENSSSCG00000017378	DHX8	6,74849E-28	27,63051004	intron_variant
ENSSSCG00000034746	CAPN8	1,48408E-08	27,67602768	intron_variant
ENSSSCG00000003917	TESK2	2,26989E-30	27,69336384	intron_variant
ENSSSCG00000034742	MECP2	9,32853E-14	27,69715587	intron_variant
ENSSSCG00000053916	-	1,82312E-19	27,70448549	upstream_gene_variant
ENSSSCG00000003368	RNF207	5,28818E-06	27,75463251	downstream_gene_variant
ENSSSCG00000004119	GRM1	3,2368E-14	27,76238101	3_prime_UTR_variant
ENSSSCG00000003773	AK5	1,86717E-06	27,78212209	intron_variant
ENSSSCG00000004027	PDE10A	2,01866E-08	27,78379885	intron_variant
ENSSSCG00000009691	C8orf74	4,08285E-22	27,79601519	upstream_gene_variant
ENSSSCG00000034964	STX8	0,000331961	27,79874214	intron_variant
ENSSSCG00000002363	VSX2	2,47577E-10	27,82604047	intron_variant
ENSSSCG00000010703	HTRA1	2,18241E-07	27,85839161	intron_variant
ENSSSCG00000008710	JAKMIP1	8,69349E-06	27,87125656	intron_variant
ENSSSCG00000005953	KCNQ3	2,74417E-19	27,88989442	intron_variant
ENSSSCG00000034768	CYTH3	6,43003E-22	27,94485056	intron_variant
ENSSSCG00000017255	ABCA5	9,58237E-08	27,96945544	intron_variant
ENSSSCG00000030603	TATDN1	4,82047E-12	27,98263584	upstream_gene_variant
ENSSSCG00000003965	-	1,73667E-11	27,9849736	intron_variant
ENSSSCG00000032741	TBC1D9	9,85468E-19	28,01830585	intron_variant
ENSSSCG00000011085	MLLT10	4,5933E-11	28,03243548	intron_variant
ENSSSCG00000004460	IBTK	6,09548E-23	28,08641975	intron_variant
ENSSSCG00000003693	MYOM1	8,11158E-16	28,1056837	intron_variant
ENSSSCG00000062369	-	2,559E-07	28,1147541	upstream_gene_variant
ENSSSCG00000017167	CYTH1	9,46272E-08	28,11947685	downstream_gene_variant
ENSSSCG00000003909	PIK3R3	2,29643E-06	28,13111546	intron_variant
ENSSSCG00000009755	AACS	6,33502E-15	28,14698761	intron_variant
ENSSSCG00000001372	GNL1	5,24366E-50	28,14798555	upstream_gene_variant
ENSSSCG00000014401	NR3C1	4,52125E-14	28,15206381	intron_variant
ENSSSCG00000015281	PLEKHA6	2,66724E-14	28,15764482	intron_variant
ENSSSCG00000002263	SLCO3A1	2,95663E-20	28,22471423	intron_variant
ENSSSCG00000017561	ABCC3	3,30324E-07	28,23879938	intron_variant
ENSSSCG00000017571	EME1	3,51675E-06	28,24275588	downstream_gene_variant
ENSSSCG00000006775	CAPZA1	1,38309E-07	28,24475193	intron_variant
ENSSSCG00000014267	RAPGEF6	1,43209E-18	28,26647037	intron_variant
ENSSSCG00000026323	FAM50B	4,93097E-08	28,27270959	downstream_gene_variant
ENSSSCG00000036341	-	9,59263E-12	28,27869225	intron_variant
ENSSSCG00000061372	-	1,43098E-14	28,2815735	intron_variant,non_coding_transcript_variant
ENSSSCG00000052842	-	3,96074E-07	28,29696288	non_coding_transcript_exon_variant

ENSSSCG00000009858	FBXO21	4,49411E-15	28,29877896	intron_variant
ENSSSCG00000013125	OSBP	1,23174E-05	28,31460674	missense_variant
ENSSSCG00000036261	CROCC2	4,08772E-06	28,33734617	intron_variant
ENSSSCG00000005934	TRAPPC9	4,79783E-07	28,36700337	intron_variant
ENSSSCG00000062991	KCNK12	1,15626E-08	28,37569129	intron_variant
ENSSSCG00000044583	LGR6	5,62727E-11	28,4167148	intron_variant
ENSSSCG00000015631	GRB10	1,06747E-26	28,52016109	intron_variant
ENSSSCG00000004022	RPS6KA2	2,24642E-07	28,5232235	intron_variant
ENSSSCG00000028531	SH3BP1	4,38288E-15	28,5244354	downstream_gene_variant
ENSSSCG00000014126	MSH3	8,79659E-11	28,52787456	intron_variant
ENSSSCG00000009029	ARHGAP10	6,83202E-09	28,55076851	intron_variant
ENSSSCG00000017877	ANKFY1	8,90973E-12	28,55210315	intron_variant
ENSSSCG00000022177	DIS3L2	1,65512E-09	28,5956139	intron_variant
ENSSSCG00000032849	MLPH	1,19745E-12	28,60730594	downstream_gene_variant
ENSSSCG00000009864	MED13L	1,44627E-17	28,68449023	intron_variant
ENSSSCG00000046071	-	5,146E-17	28,69936814	downstream_gene_variant
ENSSSCG00000016314	TRPM8	3,39357E-11	28,71127	intron_variant
ENSSSCG00000015524	FAM20B	9,65335E-07	28,72486609	intron_variant
ENSSSCG00000012813	BRCC3	1,84121E-11	28,75547659	intron_variant
ENSSSCG00000061965	-	4,04936E-13	28,75626398	upstream_gene_variant
ENSSSCG00000045502	WDFY4	3,51751E-24	28,79962415	intron_variant
ENSSSCG00000058774	-	3,44097E-17	28,8159204	non_coding_transcript_exon_variant
ENSSSCG00000008208	EIF2AK3	4,78006E-12	28,84913774	missense_variant
ENSSSCG00000009593	ROR2	1,3847E-35	28,852824	intron_variant
ENSSSCG00000056563	-	1,42947E-12	28,8833214	downstream_gene_variant
ENSSSCG00000011022	SVIL	1,7613E-06	28,88532049	intron_variant
ENSSSCG00000058263	-	2,82307E-10	28,91582492	non_coding_transcript_exon_variant
ENSSSCG00000025085	NEGR1	7,38512E-06	28,93270142	intron_variant
ENSSSCG00000021669	MARK3	2,34625E-15	28,94775679	intron_variant
ENSSSCG00000005469	KIAA1958	3,84513E-06	28,94906512	intron_variant
ENSSSCG00000016266	CAB39	5,86381E-13	28,95583504	intron_variant
ENSSSCG00000017227	FDXR	1,67422E-33	28,95796536	intron_variant
ENSSSCG00000006893	BCAR3	8,63126E-12	28,96948253	intron_variant
ENSSSCG00000010702	PLEKHA1	4,0779E-123	28,96960213	intron_variant
ENSSSCG00000003981	ZFP69B	7,98027E-14	28,99349601	intron_variant
ENSSSCG00000061048	-	1,71018E-20	29,01938089	downstream_gene_variant
ENSSSCG00000000730	PRMT8	3,91545E-18	29,02560891	upstream_gene_variant
ENSSSCG00000016110	KIAA2012	7,85395E-15	29,02612116	intron_variant
ENSSSCG00000007602	BAIAP2L1	2,37255E-14	29,07829275	intron_variant
ENSSSCG00000006636	GABPB2	1,12313E-32	29,10129691	upstream_gene_variant
ENSSSCG00000007472	BCAS4	1,41958E-09	29,15836102	intron_variant
ENSSSCG00000035093	KIF26A	1,86847E-08	29,16873108	intron_variant
ENSSSCG00000028635	-	5,27055E-43	29,16939322	intron_variant
ENSSSCG00000025295	NDFIP1	2,93616E-12	29,1938201	intron_variant
ENSSSCG00000026506	RALGAPA2	6,13866E-22	29,20864272	intron_variant
ENSSSCG00000011615	RAB7A	2,06617E-05	29,22340817	upstream_gene_variant

ENSSSCG00000003438	VPS13D	8,89933E-11	29,24152076	intron_variant
ENSSSCG00000004123	UTRN	2,74126E-38	29,32431493	intron_variant
ENSSSCG00000024189	SIL1	2,57452E-17	29,33756166	intron_variant
ENSSSCG00000037723	CFAP299	3,92072E-09	29,37168688	intron_variant
ENSSSCG00000003128	LIG1	4,31834E-13	29,37404486	intron_variant
ENSSSCG00000009412	LCP1	3,55704E-33	29,39908026	intron_variant
ENSSSCG00000016305	MROH2A	9,80924E-15	29,40648021	intron_variant
ENSSSCG00000003831	OMA1	1,13742E-29	29,42504557	intron_variant
ENSSSCG00000003744	MOCOS	3,16143E-11	29,43224062	intron_variant
ENSSSCG00000033217	-	3,13291E-15	29,47948316	intron_variant,non_coding_transcript_variant
ENSSSCG00000047137	-	1,81644E-11	29,49482192	5_prime_UTR_variant
ENSSSCG00000016258	DNER	7,06665E-17	29,50707627	intron_variant
ENSSSCG00000015380	CDCA7L	5,86112E-07	29,52476765	intron_variant
ENSSSCG00000001426	STK19	6,73046E-10	29,5328893	intron_variant
ENSSSCG00000041826	-	2,17606E-07	29,5363709	downstream_gene_variant
ENSSSCG00000002799	CNOT1	2,63221E-08	29,54585153	intron_variant
ENSSSCG00000001697	TMEM63B	3,53415E-29	29,56848489	intron_variant
ENSSSCG00000029169	MAPKAP1	3,10879E-70	29,57463589	intron_variant
ENSSSCG00000018007	MYH3	1,93118E-05	29,59359182	intron_variant
ENSSSCG00000000018	KIAA0930	8,93115E-20	29,65019947	intron_variant
ENSSSCG00000017671	RNFT1	1,01325E-08	29,65423851	intron_variant
ENSSSCG00000026344	MCM3AP	3,3868E-27	29,76620948	intron_variant
ENSSSCG00000027131	KLHDC10	9,16894E-13	29,78973928	intron_variant
ENSSSCG00000032177	MYO3B	5,64321E-13	29,80840088	intron_variant
ENSSSCG00000054176	-	4,16392E-18	29,85260035	upstream_gene_variant
ENSSSCG00000006876	PLPPR5	1,50073E-10	29,92694767	intron_variant
ENSSSCG00000060517	-	2,74287E-08	29,94553833	upstream_gene_variant
ENSSSCG00000037110	PCP4	2,0155E-13	29,98933556	intron_variant
ENSSSCG00000023028	JAM2	2,76979E-47	30,04691937	intron_variant
ENSSSCG00000005654	SPTAN1	4,74203E-07	30,05028098	intron_variant
ENSSSCG00000037168	TMEM92	1,617E-11	30,07849468	upstream_gene_variant
ENSSSCG00000026978	ROS1	1,83815E-12	30,14705882	intron_variant
ENSSSCG00000011567	-	3,17899E-15	30,14791471	intron_variant
ENSSSCG00000009683	KIF13B	4,95134E-31	30,17442031	intron_variant
ENSSSCG00000032861	NUAK1	1,82324E-10	30,17648381	intron_variant
ENSSSCG00000035949	FTO	1,76245E-10	30,19610273	intron_variant
ENSSSCG00000003423	DRAXIN	6,31394E-28	30,20737563	intron_variant
ENSSSCG00000016379	HDLBP	5,92847E-07	30,21551724	intron_variant
ENSSSCG00000034379	MAP2K3	1,12574E-13	30,23370368	intron_variant
ENSSSCG00000009666	EPHX2	3,86368E-43	30,27095263	intron_variant
ENSSSCG00000039854	TLCD4	1,32802E-20	30,27360405	intron_variant
ENSSSCG00000044700	COL4A1	8,67366E-07	30,28879016	intron_variant
ENSSSCG00000000982	TTLL8	2,94877E-39	30,29545833	intron_variant
ENSSSCG00000010811	KCTD3	6,41697E-35	30,34649204	intron_variant
ENSSSCG00000028501	-	4,57367E-16	30,35433954	upstream_gene_variant
ENSSSCG00000006903	RPAP2	1,01066E-31	30,35667959	intron_variant

ENSSSCG00000009370	FOXO1	4,22593E-11	30,35850496	downstream_gene_variant
ENSSSCG00000014321	KLHL3	7,6777E-37	30,42878141	intron_variant
ENSSSCG00000048655	-	3,99774E-23	30,43545238	upstream_gene_variant
ENSSSCG00000022029	RAP1GAP	8,27987E-16	30,44183396	downstream_gene_variant
ENSSSCG00000053413	-	5,62288E-17	30,46570407	upstream_gene_variant
ENSSSCG00000013311	KIAA1549L	4,79006E-16	30,4882721	intron_variant
ENSSSCG00000007198	ANGPT4	1,28097E-07	30,53052093	intron_variant
ENSSSCG00000033470	U1	1,51714E-09	30,57823129	upstream_gene_variant
ENSSSCG00000052075	-	6,89516E-17	30,60537116	intron_variant,non_coding_transcript_variant
ENSSSCG00000011128	ITIH2	9,78029E-29	30,60621897	upstream_gene_variant
ENSSSCG00000004955	MAP2K5	1,38199E-27	30,65675583	intron_variant
ENSSSCG00000017072	GALNT10	4,43831E-15	30,67023196	intron_variant
ENSSSCG00000054183	-	1,09774E-07	30,67787269	intron_variant,non_coding_transcript_variant
ENSSSCG00000021748	PRR5	5,1631E-13	30,69253647	intron_variant
ENSSSCG00000039171	MRTFB	6,32151E-21	30,69871459	3_prime_UTR_variant
ENSSSCG00000009374	-	2,66303E-31	30,72014282	downstream_gene_variant
ENSSSCG00000048397	-	3,57029E-16	30,7245327	intron_variant,non_coding_transcript_variant
ENSSSCG00000011788	VPS8	6,82382E-13	30,73218748	intron_variant
ENSSSCG00000014431	AFAP1L1	9,67553E-07	30,7702865	intron_variant
ENSSSCG00000011119	ECHDC3	1,47149E-13	30,77367364	intron_variant
ENSSSCG00000026940	CASP10	2,42713E-10	30,81992634	intron_variant
ENSSSCG00000012882	TESMIN	3,09125E-67	30,82683446	downstream_gene_variant
ENSSSCG00000033334	COPS7B	4,39528E-07	30,8338393	intron_variant
ENSSSCG00000017191	SRP68	1,60011E-10	30,88373846	intron_variant
ENSSSCG00000005459	ECPAS	2,53595E-17	30,9178744	intron_variant
ENSSSCG00000036812	-	1,9645E-18	30,93290988	intron_variant
ENSSSCG00000029570	PXDN	1,63777E-32	30,94319474	intron_variant
ENSSSCG00000054574	-	8,18873E-15	30,94373193	downstream_gene_variant
ENSSSCG00000052831	-	1,03317E-15	30,94624974	intron_variant,non_coding_transcript_variant
ENSSSCG00000006737	IGSF3	2,21921E-23	30,94744859	upstream_gene_variant
ENSSSCG00000012029	BACH1	2,82772E-21	30,97535087	downstream_gene_variant
ENSSSCG00000035839	TOP3A	3,93163E-12	30,98677673	intron_variant
ENSSSCG00000055472	-	8,86809E-35	31,04650669	non_coding_transcript_exon_variant
ENSSSCG00000026744	ZDHHC20	2,43806E-13	31,06719368	intron_variant
ENSSSCG00000032029	PPP2R3B	1,6352E-13	31,08666949	intron_variant
ENSSSCG00000059350	-	1,14454E-13	31,10347051	downstream_gene_variant
ENSSSCG00000014835	C2CD3	4,13091E-48	31,1404282	intron_variant
ENSSSCG00000000737	TULP3	1,23805E-18	31,1769991	intron_variant
ENSSSCG00000002901	UPK1A	1,50054E-08	31,1786253	intron_variant
ENSSSCG00000030513	CFAP74	4,25287E-33	31,2254025	upstream_gene_variant
ENSSSCG00000046312	-	9,15941E-17	31,23219373	downstream_gene_variant
ENSSSCG00000010959	NTRK2	2,52167E-12	31,23623011	intron_variant
ENSSSCG00000016569	SMO	4,94514E-14	31,31910235	intron_variant
ENSSSCG00000022777	SLC35C2	5,44677E-06	31,33802817	downstream_gene_variant
ENSSSCG00000009408	LRCH1	1,91634E-25	31,33855443	intron_variant
ENSSSCG00000016340	ASB1	5,57561E-14	31,44149534	intron_variant

ENSSSCG00000061869	-	5,19437E-08	31,45576829	intron_variant,non_coding_transcript_variant
ENSSSCG00000014094	AGGF1	1,38763E-13	31,47822733	downstream_gene_variant
ENSSSCG00000017194	TEN1	7,81976E-21	31,50095207	intron_variant
ENSSSCG00000000968	SBF1	8,63101E-56	31,51006433	downstream_gene_variant
ENSSSCG00000027774	ZFP28	1,93869E-26	31,53036577	downstream_gene_variant
ENSSSCG00000025966	EIF4H	2,46458E-12	31,55467721	upstream_gene_variant
ENSSSCG00000034444	EIF4EBP2	5,09628E-66	31,57446559	intron_variant
ENSSSCG00000037341	RNF220	3,08059E-11	31,63615561	intron_variant
ENSSSCG00000037507	BTBD9	6,16479E-12	31,66004829	intron_variant
ENSSSCG00000000014	FAM118A	2,01179E-25	31,7032873	downstream_gene_variant
ENSSSCG00000008651	DCDC2C	8,63856E-19	31,74389913	intron_variant
ENSSSCG00000054345	-	4,16662E-09	31,82165319	non_coding_transcript_exon_variant
ENSSSCG00000000006	PPARA	1,22357E-92	31,84620126	intron_variant
ENSSSCG00000006155	ZBTB10	5,78981E-18	31,94366263	3_prime_UTR_variant
ENSSSCG00000031858	KCNJ10	2,73435E-22	31,97740113	upstream_gene_variant
ENSSSCG00000000975	PANX2	7,0855E-174	32,09382694	upstream_gene_variant
ENSSSCG00000023994	ARMC9	4,54133E-24	32,11899097	missense_variant
ENSSSCG00000029326	CCNB1	2,10999E-34	32,12380053	downstream_gene_variant
ENSSSCG00000032985	GOT2	7,0701E-16	32,27656218	intron_variant
ENSSSCG00000031757	CARS2	4,6859E-23	32,30769231	intron_variant
ENSSSCG00000054135	-	2,65318E-19	32,3395812	downstream_gene_variant
ENSSSCG00000035912	ABCG5	2,92136E-32	32,34466468	intron_variant
ENSSSCG00000015072	PCSK7	1,22465E-14	32,39239239	intron_variant
ENSSSCG00000016368	FARP2	4,07586E-18	32,41487455	intron_variant
ENSSSCG00000010756	MGMT	1,85822E-10	32,45533809	intron_variant
ENSSSCG00000037269	LANCL3	2,75547E-11	32,461393	intron_variant
ENSSSCG00000035218	ADA2	7,1876E-10	32,50448296	intron_variant
ENSSSCG00000032978	-	5,33427E-14	32,54281788	intron_variant
ENSSSCG00000038622	HS3ST3A1	8,90368E-11	32,63195302	intron_variant
ENSSSCG00000017863	TRPV1	8,15392E-09	32,67922554	intron_variant
ENSSSCG00000009667	-	1,1647E-12	32,69148251	intron_variant
ENSSSCG00000006885	ALG14	3,07891E-16	32,70015328	intron_variant
ENSSSCG00000040510	KIF9	2,03863E-10	32,7071676	intron_variant
ENSSSCG00000024614	ITSN1	1,53606E-37	32,7077748	intron_variant
ENSSSCG00000025590	-	1,96633E-42	32,70795878	intron_variant
ENSSSCG00000016668	PDE1C	1,00894E-36	32,77096434	intron_variant
ENSSSCG00000039342	DNAI1	1,04252E-16	32,83657091	intron_variant
ENSSSCG00000012065	KCNJ6	3,77914E-12	32,85357357	intron_variant
ENSSSCG00000007470	RIPOR3	1,29427E-28	32,88545836	intron_variant
ENSSSCG00000060566	-	1,46796E-15	32,96191177	upstream_gene_variant
ENSSSCG00000004193	ENPP1	6,79586E-58	33,00396715	intron_variant
ENSSSCG00000026945	LRRC2	4,81542E-17	33,00467822	intron_variant
ENSSSCG00000039956	-	1,98507E-29	33,0137114	upstream_gene_variant
ENSSSCG00000025130	KLF12	9,53607E-18	33,03121283	intron_variant
ENSSSCG00000031273	IMPG2	4,27378E-24	33,04350488	intron_variant
ENSSSCG00000010494	SORBS1	6,16495E-18	33,09090909	intron_variant

ENSSSCG00000029196	DIP2B	2,95569E-19	33,11364721	intron_variant
ENSSSCG00000018019	ARHGAP44	1,55393E-15	33,26049561	intron_variant
ENSSSCG00000003811	ROR1	6,1641E-59	33,27782665	intron_variant
ENSSSCG00000017346	EFTUD2	1,57515E-20	33,29193672	intron_variant
ENSSSCG00000033193	TPO	6,52018E-11	33,2951477	downstream_gene_variant
ENSSSCG00000040130	NALF1	2,70048E-11	33,31162488	intron_variant
ENSSSCG00000022636	DENND5B	6,74919E-08	33,35227273	intron_variant
ENSSSCG00000011026	ARHGAP12	6,7841E-14	33,37363294	intron_variant
ENSSSCG00000021791	SENP7	1,47987E-30	33,4331116	intron_variant
ENSSSCG00000033228	SPAG9	2,44994E-10	33,43495935	intron_variant
ENSSSCG00000029502	NAXD	6,38888E-16	33,45562944	intron_variant
ENSSSCG00000006750	SYCP1	1,5079E-27	33,52040816	intron_variant
ENSSSCG00000049847	-	1,25249E-17	33,52197471	missense_variant
ENSSSCG00000002506	VRK1	2,1248E-31	33,6637931	intron_variant
ENSSSCG00000000970	PPP6R2	1,5014E-37	33,66679715	intron_variant
ENSSSCG00000015923	NOSTRIN	1,11252E-60	33,67097515	intron_variant
ENSSSCG00000033015	B3GNT7	9,98366E-27	33,69316739	downstream_gene_variant
ENSSSCG00000029633	USP24	1,54863E-08	33,72093023	intron_variant
ENSSSCG00000034156	GTF2F2	1,86351E-15	33,76248587	intron_variant
ENSSSCG00000003136	CYTH2	1,95043E-21	33,76729653	intron_variant
ENSSSCG00000058033	HINFP	5,82236E-08	33,787476	missense_variant
ENSSSCG00000005609	GARNL3	1,86047E-07	33,82041652	intron_variant
ENSSSCG00000021994	U6	2,0838E-27	33,85044292	upstream_gene_variant
ENSSSCG00000024592	ANKRD17	1,91032E-10	33,86006975	intron_variant
ENSSSCG00000017988	CCDC42	4,40948E-22	33,86181229	intron_variant
ENSSSCG00000007478	ATP9A	9,9323E-11	33,96226415	intron_variant
ENSSSCG00000008222	CHMP3	1,70146E-35	33,96308609	downstream_gene_variant
ENSSSCG00000061469	-	6,20302E-34	33,96574353	downstream_gene_variant
ENSSSCG00000033636	AFMID	3,3895E-10	34,06957828	intron_variant
ENSSSCG00000010514	RRP12	1,85655E-17	34,08963585	intron_variant
ENSSSCG00000056251	CCL22	8,41456E-28	34,12729451	3_prime_UTR_variant
ENSSSCG00000008689	ZFYVE28	4,47403E-11	34,17983934	intron_variant
ENSSSCG00000001816	BLM	3,13973E-14	34,18501048	intron_variant
ENSSSCG00000014394	RNF14	2,89542E-09	34,36249627	upstream_gene_variant
ENSSSCG00000023890	ATG2A	1,5971E-19	34,62610972	intron_variant
ENSSSCG00000021888	ZNF135	4,77267E-24	34,68137255	upstream_gene_variant
ENSSSCG00000001567	CPNE5	1,45813E-17	34,75863504	intron_variant
ENSSSCG00000033731	SLC35B1	4,4445E-13	34,78337443	3_prime_UTR_variant
ENSSSCG00000010521	UBTD1	1,03218E-13	34,79124121	intron_variant
ENSSSCG00000007485	BCAS1	2,43659E-07	34,81182796	intron_variant
ENSSSCG00000004826	SELENOS	6,43829E-19	34,8269322	intron_variant
ENSSSCG00000009293	-	2,04893E-58	34,92731306	intron_variant
ENSSSCG00000025534	CSE1L	5,28426E-06	34,93965664	intron_variant
ENSSSCG00000006911	TGFBR3	1,22304E-16	34,95699935	intron_variant
ENSSSCG00000011056	FRMD4A	1,70548E-41	34,98711552	intron_variant
ENSSSCG00000035852	CSNK1D	9,79322E-14	35,06636527	intron_variant

ENSSSCG00000012271	RBM10	1,26428E-23	35,12399084	intron_variant
ENSSSCG00000015537	XPR1	8,6123E-14	35,13402906	intron_variant
ENSSSCG00000006183	SBSPON	1,85551E-42	35,17099863	intron_variant
ENSSSCG00000032644	HLX	7,1549E-09	35,18821604	downstream_gene_variant
ENSSSCG00000030547	PIP5K1A	3,8157E-11	35,32555642	upstream_gene_variant
ENSSSCG00000007422	DNTTIP1	5,1692E-10	35,3347136	intron_variant
ENSSSCG00000017168	SEPTIN9	6,69247E-09	35,40131412	intron_variant
ENSSSCG00000009664	PTK2B	5,6812E-127	35,53787419	intron_variant
ENSSSCG00000041310	-	2,24799E-11	35,59947299	non_coding_transcript_exon_variant
ENSSSCG00000055339	-	1,50011E-19	35,64040821	upstream_gene_variant
ENSSSCG00000047003	-	1,04095E-19	35,72241414	intron_variant
ENSSSCG00000016423	DPP6	1,62253E-17	35,8731888	intron_variant
ENSSSCG00000017069	LARP1	2,50197E-26	35,88160036	intron_variant
ENSSSCG00000012112	-	3,16181E-37	35,9103398	intron_variant
ENSSSCG00000021514	OCLN	8,85388E-17	35,9370725	3_prime_UTR_variant
ENSSSCG00000051819	MYCBP	9,34633E-14	35,96302891	downstream_gene_variant
ENSSSCG00000016545	CHCHD3	7,62258E-48	35,98884378	intron_variant
ENSSSCG00000033037	-	1,19954E-35	35,99179299	intron_variant
ENSSSCG00000041764	CACNA1C	2,81746E-22	36,03759553	intron_variant
ENSSSCG00000003286	-	1,06997E-10	36,07474086	downstream_gene_variant
ENSSSCG00000007166	PTPRA	3,31405E-14	36,08804705	intron_variant
ENSSSCG00000029251	MAP4	1,64059E-17	36,17700969	intron_variant
ENSSSCG00000002266	CHD2	7,18161E-56	36,17986357	intron_variant
ENSSSCG00000027898	ATP2B1	6,93932E-19	36,56554713	intron_variant
ENSSSCG00000011363	PRKAR2A	2,70102E-17	36,59722222	intron_variant
ENSSSCG00000025912	URI1	2,2415E-104	36,67424075	intron_variant
ENSSSCG00000035581	SUGCT	2,34168E-25	36,77192149	intron_variant
ENSSSCG00000055311	-	1,85132E-31	36,80536913	non_coding_transcript_exon_variant
ENSSSCG00000032086	ZNF583	1,74271E-18	36,95621737	upstream_gene_variant
ENSSSCG00000040449	ABHD12	2,90947E-45	37,00411488	intron_variant
ENSSSCG00000016843	CPLANE1	9,53918E-16	37,09007887	intron_variant
ENSSSCG00000003428	MTHFR	9,6198E-15	37,38927063	downstream_gene_variant
ENSSSCG00000017993	NTN1	1,68934E-24	37,44924339	intron_variant
ENSSSCG00000037530	-	6,8237E-17	37,53197642	intron_variant
ENSSSCG00000021805	GALNTL6	1,27809E-14	37,71589311	intron_variant
ENSSSCG00000002451	RIN3	6,52076E-17	37,74011299	intron_variant
ENSSSCG00000008066	ZNF484	1,16526E-47	37,76863862	intron_variant
ENSSSCG00000007188	-	9,23147E-23	37,77720695	3_prime_UTR_variant
ENSSSCG00000016521	DGKI	2,36237E-10	37,85425101	intron_variant
ENSSSCG00000029231	-	3,22665E-55	37,97041624	downstream_gene_variant
ENSSSCG00000033329	SLC47A1	9,62282E-28	38,01016703	upstream_gene_variant
ENSSSCG00000014108	BHMT	6,25407E-22	38,03588991	intron_variant
ENSSSCG00000060636	-	9,69093E-11	38,16568047	intron_variant
ENSSSCG00000033192	KLK9	8,9655E-155	38,28585058	upstream_gene_variant
ENSSSCG00000022034	SMYD3	2,0552E-33	38,29274977	intron_variant
ENSSSCG00000015538	KIAA1614	3,37425E-29	38,30352038	intron_variant

ENSSSCG00000000755	ERC1	1,23048E-77	38,45280903	intron_variant
ENSSSCG00000012832	-	6,59649E-42	38,49723867	non_coding_transcript_exon_variant
ENSSSCG00000015952	HAT1	1,03008E-11	38,54068117	intron_variant
ENSSSCG00000035252	SMIM17	8,69818E-59	38,60829604	intron_variant
ENSSSCG00000034040	TMEM11	4,78665E-21	38,61336032	intron_variant
ENSSSCG00000058491	-	5,93213E-54	38,76026273	non_coding_transcript_exon_variant
ENSSSCG00000000003	TTC38	6,13251E-61	38,8392834	upstream_gene_variant
ENSSSCG00000059216	-	1,84664E-13	38,86328725	downstream_gene_variant
ENSSSCG00000057805	-	1,26759E-13	39,05502392	non_coding_transcript_exon_variant
ENSSSCG00000007073	ISM1	1,80141E-21	39,10950908	intron_variant
ENSSSCG00000062725	-	1,76388E-10	39,13305238	intron_variant
ENSSSCG00000018147	ssc-mir-365-2	1,22497E-12	39,27404148	downstream_gene_variant
ENSSSCG00000029553	CCDC57	1,20365E-30	39,28115565	intron_variant
ENSSSCG00000057616	-	5,1305E-26	39,38025816	upstream_gene_variant
ENSSSCG00000010835	AIDA	1,46029E-34	39,55638221	intron_variant
ENSSSCG00000037997	-	1,81266E-39	39,56867422	intron_variant,non_coding_transcript_variant
ENSSSCG00000061457	-	5,18963E-93	39,73256379	non_coding_transcript_exon_variant
ENSSSCG00000002457	ITPK1	6,98839E-25	39,781491	intron_variant
ENSSSCG00000035658	VPS36	4,39863E-18	39,79219678	intron_variant
ENSSSCG00000031398	-	9,21938E-18	40,08320277	upstream_gene_variant
ENSSSCG00000010512	SLIT1	3,01438E-65	40,09152783	intron_variant
ENSSSCG00000014127	RASGRF2	4,89417E-14	40,13964235	intron_variant
ENSSSCG00000055601	-	2,24205E-32	40,15363096	upstream_gene_variant
ENSSSCG00000038760	ANKRD40	4,39415E-17	40,20950294	upstream_gene_variant
ENSSSCG00000040786	SPTBN1	9,4119E-32	40,3793119	intron_variant
ENSSSCG00000049630	DEPTOR	1,82673E-31	40,45600816	intron_variant
ENSSSCG00000059405	-	1,96168E-10	40,60198714	upstream_gene_variant
ENSSSCG00000006391	ATP1A2	3,63152E-14	40,86551194	intron_variant
ENSSSCG00000001403	LTA	7,3586E-51	40,92718227	3_prime_UTR_variant
ENSSSCG00000040038	ZNF8	9,46842E-20	41,1780852	downstream_gene_variant
ENSSSCG00000037232	SNX29	1,1538E-15	41,21810207	intron_variant
ENSSSCG00000022290	UMODL1	9,46809E-18	41,2202381	intron_variant
ENSSSCG00000038763	CERK	1,67901E-75	41,24423649	intron_variant
ENSSSCG00000014097	PDE8B	2,14967E-16	41,30075793	intron_variant
ENSSSCG00000005706	ABL1	1,86884E-14	41,42857143	intron_variant
ENSSSCG00000007721	GTF2I	1,63246E-35	41,49622637	intron_variant
ENSSSCG00000015670	GTDC1	7,57641E-34	41,78905207	intron_variant
ENSSSCG00000038226	-	2,30173E-22	41,80225715	intron_variant
ENSSSCG00000007155	DNAAF9	6,83802E-32	41,94565759	intron_variant
ENSSSCG00000038135	-	7,46972E-69	41,95988441	intron_variant
ENSSSCG00000003269	LENG9	8,8009E-23	42,01288245	downstream_gene_variant
ENSSSCG00000060319	-	4,8908E-102	42,09280601	downstream_gene_variant
ENSSSCG00000063338	-	9,95396E-18	42,23347042	downstream_gene_variant
ENSSSCG00000034528	TGM6	8,85098E-71	42,42432129	downstream_gene_variant
ENSSSCG00000012103	-	1,7383E-115	42,4376052	intron_variant
ENSSSCG00000033351	-	4,04181E-17	42,49241763	downstream_gene_variant

ENSSSCG00000021310	LIN9	6,73761E-66	42,88366984	intron_variant
ENSSSCG00000008501	VIT	7,75173E-69	42,99902768	intron_variant
ENSSSCG00000009839	CIT	5,67364E-21	43,24423963	intron_variant
ENSSSCG00000063019	-	1,17547E-77	43,25904974	downstream_gene_variant
ENSSSCG00000024633	JAKMIP3	1,42664E-17	43,36472844	missense_variant
ENSSSCG00000023054	IDO2	3,9022E-30	43,39738152	5_prime_UTR_variant
ENSSSCG00000035527	-	1,42606E-30	43,56228513	intron_variant
ENSSSCG00000033259	ARMH1	7,40319E-28	43,76351839	intron_variant
ENSSSCG00000007167	VPS16	1,76763E-68	43,95892355	intron_variant
ENSSSCG00000003918	TOE1	2,43176E-31	44,20862666	upstream_gene_variant
ENSSSCG00000000062	CSDC2	4,47861E-57	44,76480836	intron_variant
ENSSSCG00000052878	GPR32	8,48793E-34	44,8927477	intron_variant
ENSSSCG00000038009	-	1,15951E-27	45,00474168	downstream_gene_variant
ENSSSCG00000030668	UQCC2	1,90369E-38	45,17878666	intron_variant
ENSSSCG00000003119	ZNF541	3,66767E-28	45,46929443	intron_variant
ENSSSCG00000057382	-	1,49706E-61	45,87576646	non_coding_transcript_exon_variant
ENSSSCG00000017219	HID1	4,04026E-26	45,95881505	intron_variant
ENSSSCG00000005951	TMEM71	8,91783E-37	46,16348564	downstream_gene_variant
ENSSSCG00000026498	R3HDM2	5,51117E-41	46,35809313	intron_variant
ENSSSCG00000051568	-	8,12257E-24	46,36070853	downstream_gene_variant
ENSSSCG00000015711	DPP10	5,9896E-112	46,57557042	intron_variant
ENSSSCG00000003124	CRX	9,18375E-19	46,64393939	upstream_gene_variant
ENSSSCG00000038327	-	1,2033E-17	47,04892415	intron_variant
ENSSSCG00000001826	CFAP100	2,99041E-23	47,53219793	intron_variant
ENSSSCG00000001078	MBOAT1	6,50335E-35	47,53224685	intron_variant
ENSSSCG00000042769	-	5,3894E-119	47,56810907	downstream_gene_variant
ENSSSCG00000003160	NTF4	6,41048E-33	47,88568257	downstream_gene_variant
ENSSSCG00000032284	CCND3	3,30495E-82	47,96227948	intron_variant
ENSSSCG00000009268	CRYL1	1,95739E-25	48,25681492	intron_variant
ENSSSCG00000017553	KAT7	1,19682E-16	48,28390001	intron_variant
ENSSSCG00000010699	ATE1	3,0171E-22	48,70558148	upstream_gene_variant
ENSSSCG00000006619	SNX27	7,78636E-37	48,96066044	intron_variant
ENSSSCG00000006614	THEM5	7,9545E-128	49,08249158	intron_variant
ENSSSCG00000050390	-	2,01109E-57	49,11955062	downstream_gene_variant
ENSSSCG00000010912	KIF14	5,67148E-16	49,12124978	downstream_gene_variant
ENSSSCG00000015244	APLP2	3,36004E-71	49,17680369	intron_variant
ENSSSCG00000011456	CHDH	9,1897E-274	49,38611209	upstream_gene_variant
ENSSSCG00000020791	SNORD23	6,77885E-27	49,60637527	downstream_gene_variant
ENSSSCG00000012884	PPP6R3	4,15144E-20	49,84186657	downstream_gene_variant
ENSSSCG00000007477	NFATC2	1,96616E-15	50,1269304	intron_variant
ENSSSCG00000000918	EPYC	1,0546E-18	50,35650624	upstream_gene_variant
ENSSSCG00000036274	-	1,05325E-24	50,91389013	intron_variant
ENSSSCG00000003114	DHX34	1,95873E-24	51,46031746	intron_variant
ENSSSCG00000035785	-	1,13657E-47	52,11222352	intron_variant,non_coding_transcript_variant
ENSSSCG00000058292	-	2,37324E-46	52,18918863	upstream_gene_variant
ENSSSCG00000003218	MYBPC2	5,9437E-114	52,24352519	intron_variant

ENSSSCG00000022080	RHOA	2,76397E-48	53,03952991	intron_variant
ENSSSCG00000003157	LIN7B	6,22069E-27	53,7699293	upstream_gene_variant
ENSSSCG00000058639	-	4,82721E-41	53,7944664	intron_variant
ENSSSCG00000003123	BSPH1	7,2209E-108	54,56038949	intron_variant
ENSSSCG00000009680	EXTL3	5,23436E-31	55,76816653	intron_variant
ENSSSCG00000038535	ARSB	4,48836E-87	55,8302456	intron_variant
ENSSSCG00000040922	IP6K2	3,55555E-31	57,39868468	intron_variant
ENSSSCG00000026689	-	0	58,88652076	intron_variant
ENSSSCG00000006717	PHGDH	5,79086E-64	59,19911568	intron_variant
ENSSSCG00000050385	-	6,20593E-49	59,64259213	downstream_gene_variant
ENSSSCG00000045200	-	1,38828E-41	59,6892964	upstream_gene_variant
ENSSSCG00000010723	ACADSB	9,00618E-55	63,0630259	intron_variant
ENSSSCG00000003133	GRIN2D	4,94574E-58	63,11848762	intron_variant
ENSSSCG00000029289	CST9L	2,0488E-96	63,29036219	downstream_gene_variant
ENSSSCG00000003139	BCAT2	3,5695E-207	64,50532725	intron_variant
