# Supplementary material for: Changes in DNA Methylation and mRNA Expression in Lung Tissue after Long-Term Supplementation with an Increased Dose of Cholecalciferol
Source: Int J Mol Sci. 2023 Dec 29;25(1):464. doi: 10.3390/ijms25010464 (PMC10778667; doi:10.3390/ijms25010464)
Supplement: Supplementary file 1 [file ijms-25-00464-s001.zip › Supplementary Material Table S3.pdf]

Supplementary Material Table S3. Statistics of mRNA library sequencing results.

Sample	No. of raw reads	No. of filtered reads	% of uniquely mapped reads	No. of uniquely mapped reads
3II	11496566	11488281	89,43	10273612
9I	12 121 290	12 000 077	87,44	10492867
17I	11591508	11582134	88,07	10199993
19II	11758736	11750855	89,03	10461987
27II	11497680	11489917	88,04	10115440
33I	11466406	11458489	88,64	10157368
41I	12128355	12120476	87,83	10645346
51II	10193864	10186483	87,77	8940776
59II	10484849	10477585	88,89	9313629
**Average**	**11327246**	**11319278**	**88,348889**	**10066780**
