# Supplementary material for: Changes in DNA Methylation and mRNA Expression in Lung Tissue after Long-Term Supplementation with an Increased Dose of Cholecalciferol
Source: Int J Mol Sci. 2023 Dec 29;25(1):464. doi: 10.3390/ijms25010464 (PMC10778667; doi:10.3390/ijms25010464)
Supplement: Supplementary file 1 [file ijms-25-00464-s001.zip › Supplementary Material Table S4.pdf]

Supplementary Material Table S4. Changes in gene expression identified in animals receiving an increased dose of cholecalciferol.

Ensembl ID	Gene name	padj	log2FoldChange
ENSSSCG00000029558	EXTL1	0,038732754	-4,039570718
ENSSSCG00000057577	-	0,007759808	-3,619492816
ENSSSCG00000018197	-	0,046793049	-3,423124255
ENSSSCG00000017998	GLP2R	0,025709055	-3,36010522
ENSSSCG00000016746	NPC1L1	0,036556591	-3,279430237
ENSSSCG00000042623	-	0,007847437	-3,050573382
ENSSSCG00000028896	DIO1	0,031212423	-2,878830124
ENSSSCG00000015590	FLVCR1	0,04208461	-2,877124374
ENSSSCG00000057480	-	0,036381088	-2,851059357
ENSSSCG00000000892	HAL	0,007759808	-2,831315471
ENSSSCG00000022009	DDC	0,007759808	-2,827721954
ENSSSCG00000044191	-	0,047472057	-2,817342084
ENSSSCG00000049319	-	0,04203598	-2,776425339
ENSSSCG00000010065	GSTT4	0,031212423	-2,745523962
ENSSSCG00000017226	FADS6	0,009069888	-2,657149523
ENSSSCG00000053426	-	0,031212423	-2,579890865
ENSSSCG00000011397	SLC38A3	0,033512727	-2,572004276
ENSSSCG00000055939	-	0,012490964	-2,558527905
ENSSSCG00000018715	Y_RNA	0,047261642	-2,553918376
ENSSSCG00000037234	CLDN2	0,031212423	-2,534153857
ENSSSCG00000004643	SLC27A2	0,034854441	-2,526253181
ENSSSCG00000028031	HDAC11	0,048438501	-2,518169541
ENSSSCG00000001901	CYP1A2	0,018436773	-2,434211111
ENSSSCG00000003901	FAAH	0,04182479	-2,430620967
ENSSSCG00000044637	-	0,025709055	-2,426349698
ENSSSCG00000046051	-	0,007759808	-2,407600958
ENSSSCG00000016199	CYP27A1	0,031212423	-2,389975479
ENSSSCG00000057063	-	0,031212423	-2,387621704
ENSSSCG00000035629	CA5A	0,018436773	-2,387558944
ENSSSCG00000013767	PALM3	0,031212423	-2,351717186
ENSSSCG00000017383	-	0,011640673	-2,346152324
ENSSSCG00000034102	-	0,036857476	-2,341247282
ENSSSCG00000034598	H2AC2 -	0,042989986	-2,335129846
ENSSSCG00000021938	UPP2	0,018436773	-2,326818479
ENSSSCG00000041461	-	0,007759808	-2,325627462
ENSSSCG00000033207	SULT2A1	0,031212423	-2,320221083
ENSSSCG00000062487	KRT5	0,047472057	-2,314946701
ENSSSCG00000047427	-	0,031212423	-2,303061239
ENSSSCG00000058048	TTPA	0,018436773	-2,296666046
ENSSSCG00000024476	CES3	0,031212423	-2,267267138
ENSSSCG00000023301	F13B	0,031212423	-2,251403167
ENSSSCG00000014108	BHMT	0,031212423	-2,227394874

ENSSSCG00000012911	CARNS1	0,019990714	-2,225432386
ENSSSCG00000026453	ACSM5	0,033932908	-2,218808005
ENSSSCG00000026532	DNASE2B	0,039420853	-2,215686006
ENSSSCG00000014321	KLHL3	0,007759808	-2,213466744
ENSSSCG00000028623	RARRES1	0,025709055	-2,181260444
ENSSSCG00000001411	APOM	0,037453852	-2,175390097
ENSSSCG00000001823	UROC1	0,031212423	-2,174751336
ENSSSCG00000047163	-	0,031212423	-2,173287541
ENSSSCG00000020491	-	0,031212423	-2,172717246
ENSSSCG00000011128	ITIH2	0,018436773	-2,164157583
ENSSSCG00000055043	-	0,033932908	-2,156732126
ENSSSCG00000002355	ENTPD5	0,021294362	-2,143384781
ENSSSCG00000037509	EIF1AY	0,03699089	-2,117846267
ENSSSCG00000055196	-	0,034854441	-2,114526257
ENSSSCG00000017311	MAPT	0,039420853	-2,11220501
ENSSSCG00000052947	-	0,031212423	-2,10140178
ENSSSCG00000006987	SLC7A2	0,031212423	-2,093050708
ENSSSCG00000016379	HDLBP	0,031212423	-2,089295551
ENSSSCG00000011802	KNG1	0,031212423	-2,085192123
ENSSSCG00000028115	ALDH8A1	0,041029725	-2,076109547
ENSSSCG00000015799	KLKB1	0,04208461	-2,073129298
ENSSSCG00000003835	C8A	0,032666994	-2,068517983
ENSSSCG00000006719	-	0,049312314	-2,061823832
ENSSSCG00000011799	AHSG	0,031212423	-2,049714716
ENSSSCG00000050719	-	0,039420853	-2,03178731
ENSSSCG00000002009	PCK2	0,034854441	-2,02041364
ENSSSCG00000015901	GRB14	0,034854441	-2,015712536
ENSSSCG00000040910	APOH	0,031212423	-2,013285157
ENSSSCG00000055714	-	0,047472057	-1,995222257
ENSSSCG00000010545	CPN1	0,036556591	-1,988042163
ENSSSCG00000034188	-	0,033767671	-1,987484193
ENSSSCG00000005485	AMBP	0,031212423	-1,984124263
ENSSSCG00000020680	CLDN14	0,03699089	-1,979268789
ENSSSCG00000013252	F2	0,031212423	-1,969124708
ENSSSCG00000038558	GOLPH3L	0,036347524	-1,968789602
ENSSSCG00000039095	-	0,031212423	-1,968515731
ENSSSCG00000055459	-	0,049312314	-1,956133055
ENSSSCG00000013150	-	0,036556591	-1,956020479
ENSSSCG00000023415	-	0,031212423	-1,946358291
ENSSSCG00000027801	VTN	0,039420853	-1,940476728
ENSSSCG00000006040	DPYS	0,048792299	-1,91067194
ENSSSCG00000010337	MAT1A	0,031212423	-1,909057939
ENSSSCG00000009179	MTTP	0,034854441	-1,895281389
ENSSSCG00000052219	-	0,04208461	-1,889890218
ENSSSCG00000011640	TF	0,031212423	-1,886845382
ENSSSCG00000000875	NR1H4	0,046793049	-1,885563601

ENSSSCG00000023320	-	0,031403125	-1,883826633
ENSSSCG00000031698	GFUS	0,031212423	-1,880738316
ENSSSCG00000010479	RBP4	0,031212423	-1,872835114
ENSSSCG00000061364	-	0,036556591	-1,868611762
ENSSSCG00000035336	-	0,031212423	-1,862687934
ENSSSCG00000002993	-	0,031212423	-1,861800825
ENSSSCG00000010291	PLA2G12B	0,031403125	-1,856783853
ENSSSCG00000002623	-	0,031212423	-1,842879032
ENSSSCG00000057522	CDO1	0,018436773	-1,841555349
ENSSSCG00000016861	C6	0,033932908	-1,833258718
ENSSSCG00000027275	HHLA2	0,047261642	-1,831901581
ENSSSCG00000009182	-	0,03699089	-1,83046303
ENSSSCG00000051968	-	0,038056327	-1,827202225
ENSSSCG00000014187	NUDT12	0,038510684	-1,823151701
ENSSSCG00000037268	APCS	0,034854441	-1,808968878
ENSSSCG00000010431	A1CF	0,031212423	-1,801810081
ENSSSCG00000052216	-	0,04208461	-1,798075824
ENSSSCG00000063249	FABP1	0,036381088	-1,798034483
ENSSSCG00000039276	UGT2B31	0,031212423	-1,796609082
ENSSSCG00000011800	FETUB	0,037546235	-1,796481174
ENSSSCG00000027854	HSD17B6	0,042803821	-1,795617304
ENSSSCG00000036572	BCO1	0,034854441	-1,794658522
ENSSSCG00000007858	-	0,018436773	-1,793584662
ENSSSCG00000024402	-	0,049248052	-1,793255264
ENSSSCG00000027439	HAO1	0,04208461	-1,79021217
ENSSSCG00000003857	ZYG11B	0,034854441	-1,787938779
ENSSSCG00000056835	-	0,039420853	-1,787845279
ENSSSCG00000046622	-	0,031212423	-1,784732449
ENSSSCG00000030033	ACSM4	0,032666994	-1,769112778
ENSSSCG00000010116	SLC25A1	0,03699089	-1,766973828
ENSSSCG00000006403	CRP	0,041199712	-1,766121159
ENSSSCG00000015493	SERPINC1	0,046793049	-1,748953965
ENSSSCG00000001732	MMUT	0,03699089	-1,735936904
ENSSSCG00000003086	-	0,031212423	-1,727411671
ENSSSCG00000036208	SHMT1	0,033512727	-1,720198406
ENSSSCG00000014110	DMGDH	0,049312314	-1,716764597
ENSSSCG00000008998	-	0,039980977	-1,703212185
ENSSSCG00000008997	FGB	0,04208461	-1,691209412
ENSSSCG00000021998	-	0,034854441	-1,667969226
ENSSSCG00000010543	ABCC2	0,031403125	-1,665425606
ENSSSCG00000024314	FGG	0,031212423	-1,664140585
ENSSSCG00000002494	CLMN	0,04208461	-1,654972211
ENSSSCG00000002633	GAS8	0,023432223	-1,652659683
ENSSSCG00000005393	PLPPR1	0,03151148	-1,648258547
ENSSSCG00000008595	APOB	0,036857476	-1,641711717
ENSSSCG00000009184	ADH4	0,036347524	-1,63998158

ENSSSCG00000063121	-	0,031212423	-1,637565206
ENSSSCG00000029515	PON3	0,049312314	-1,630835447
ENSSSCG00000009413	CPB2	0,038510684	-1,616172782
ENSSSCG00000036274	-	0,047472057	-1,60332826
ENSSSCG00000062662	-	0,036857476	-1,595993205
ENSSSCG00000022351	GSTO1	0,039980977	-1,592850652
ENSSSCG00000016522	PTN	0,042989986	-1,589708151
ENSSSCG00000004672	GATM	0,031212423	-1,583880312
ENSSSCG00000001107	SLC17A1	0,04203598	-1,572225119
ENSSSCG00000004607	PYGO1	0,031212423	-1,562022528
ENSSSCG00000023084	ATP2B2	0,031212423	-1,553180838
ENSSSCG00000038300	ALDOB	0,047472057	-1,549682762
ENSSSCG00000026597	SLC38A4	0,047472057	-1,541801516
ENSSSCG00000062921	-	0,036556591	-1,539542481
ENSSSCG00000029275	PPARGC1A	0,03699089	-1,524629879
ENSSSCG00000029606	AOX1	0,031212423	-1,519609215
ENSSSCG00000005287	PSAT1	0,032793814	-1,511168455
ENSSSCG00000060395	-	0,034854441	-1,493305052
ENSSSCG00000048082	-	0,047472057	-1,49244024
ENSSSCG00000008935	-	0,039980977	-1,477529658
ENSSSCG00000037035	UVSSA	0,044542956	-1,467977782
ENSSSCG00000008948	ALB	0,047472057	-1,466127753
ENSSSCG00000040989	GPRC5C	0,039420853	-1,462312689
ENSSSCG00000038394	PAIP2B	0,03699089	-1,457556827
ENSSSCG00000005701	ASS1	0,040737182	-1,449372568
ENSSSCG00000005398	-	0,045693659	-1,44441252
ENSSSCG00000007507	PCK1	0,036857476	-1,424272129
ENSSSCG00000060615	TUBA8	0,03699089	-1,291041184
ENSSSCG00000035429	HJV	0,04208461	-1,289653694
ENSSSCG00000028996	ALDH1A1	0,036572705	-1,249695637
ENSSSCG00000020706	-	0,047472057	-1,210805287
ENSSSCG00000040904	CLDN1	0,036556591	-1,093657908
ENSSSCG00000023267	SCN1B	0,046793049	-1,031782009
ENSSSCG00000009290	MIPEP	0,032899246	-1,02139637
ENSSSCG00000002452	LGMN	0,032858154	0,927887653
ENSSSCG00000011859	HEG1	0,031212423	0,969753214
ENSSSCG00000053422	THBD	0,044177693	1,047124451
ENSSSCG00000016434	RHEB	0,047472057	1,083948196
ENSSSCG00000005452	C9orf152	0,04208461	1,112137106
ENSSSCG00000030053	ARCN1	0,047472057	1,117185117
ENSSSCG00000045471	-	0,03699089	1,121154693
ENSSSCG00000005453	-	0,036556591	1,150484278
ENSSSCG00000009164	CISD2	0,043136182	1,208102971
ENSSSCG00000051245	-	0,037374454	1,252101644
ENSSSCG00000000862	GNPTAB	0,031212423	1,293467665
ENSSSCG00000023972	DRAM1	0,031212423	1,297314313

ENSSSCG00000000600	EPS8	0,031212423	1,315885508
ENSSSCG00000035820	TXNDC17	0,031212423	1,349583921
ENSSSCG00000009240	-	0,043136182	1,364815101
ENSSSCG00000017336	HEXIM1	0,007759808	1,399686247
ENSSSCG00000009140	CASP6	0,03699089	1,418107717
ENSSSCG00000001063	MYLIP	0,036857476	1,524854267
ENSSSCG00000029160	-	0,018436773	1,582776715
ENSSSCG00000022230	CD9	0,007759808	1,643464523
ENSSSCG00000053185	-	0,025709055	1,65162493
ENSSSCG00000013391	PSMA1	0,039420853	1,658468844
ENSSSCG00000004142	CITED2	0,039420853	1,695387168
ENSSSCG00000005055	LGALS3	0,034854441	1,70440655
ENSSSCG00000052861	-	0,033512727	1,798557112
ENSSSCG00000017617	SCPEP1	0,039420853	3,090991219
ENSSSCG00000009474	SCEL	0,018436773	3,447755964
